# Unipolar Peptidoglycan Synthesis in the *Rhizobiales* Requires an Essential Class A Penicillin-Binding Protein

**DOI:** 10.1128/mBio.02346-21

**Published:** 2021-09-21

**Authors:** Michelle A. Williams, Alena Aliashkevich, Elizaveta Krol, Erkin Kuru, Jacob M. Bouchier, Jonathan Rittichier, Yves V. Brun, Michael S. VanNieuwenhze, Anke Becker, Felipe Cava, Pamela J. B. Brown

**Affiliations:** a Division of Biological Sciences, University of Missouri, Columbia, Missouri, USA; b Department of Molecular Biology, Laboratory for Molecular Infection Medicine Sweden (MIMS), Centre for Microbial Research, Umeå University, Umeå, Sweden; c Center for Synthetic Microbiology (SYNMIKRO), Philipps University Marburg, Marburg, Germany; d Department of Biology, Philipps University Marburg, Marburg, Germany; e Department of Chemistry, Indiana University, Bloomington, Indiana, USA; f Département de Microbiologie, Infectiologie et Immunologie, Faculté de Médecine, Université de Montréal, Montreal, Quebec, Canada; University of Massachusetts Amherst

**Keywords:** *Agrobacterium tumefaciens*, *Rhizobiales*, cell envelope, cell wall, penicillin-binding proteins, peptidoglycan, polar growth

## Abstract

Members of the *Rhizobiales* are polarly growing bacteria that lack homologs of the canonical Rod complex. To investigate the mechanisms underlying polar cell wall synthesis, we systematically probed the function of cell wall synthesis enzymes in the plant pathogen Agrobacterium tumefaciens. The development of fluorescent d-amino acid dipeptide (FDAAD) probes, which are incorporated into peptidoglycan by penicillin-binding proteins in A. tumefaciens, enabled us to monitor changes in growth patterns in the mutants. Use of these fluorescent cell wall probes and peptidoglycan compositional analysis demonstrate that a single class A penicillin-binding protein is essential for polar peptidoglycan synthesis. Furthermore, we find evidence of an additional mode of cell wall synthesis that requires ld-transpeptidase activity. Genetic analysis and cell wall targeting antibiotics reveal that the mechanism of unipolar growth is conserved in *Sinorhizobium* and Brucella. This work provides insights into unipolar peptidoglycan biosynthesis employed by the *Rhizobiales* during cell elongation.

## INTRODUCTION

Our current understanding of peptidoglycan (PG) assembly in rod-shaped bacteria stems largely from investigations conducted using well-known model species such as Escherichia coli and Bacillus subtilis, which incorporate new cell wall material along the lateral sidewalls of the cell body. Expanding our studies of cell wall synthesis to include diverse species, with alternative modes of elongation, is an important step in unveiling the mechanisms of how and why bacteria evolve novel growth modes and generate innovative morphologies. It had, for example, long been assumed that all rod-shaped bacteria employed the same growth strategy; however, unipolar growth is widespread among rod-shaped bacteria in the alphaproteobacterial order *Rhizobiales*, suggesting diversification of growth strategies (1). The *Rhizobiales* comprise diverse bacteria with respect to both their cellular morphology and their environmental niches (2, 3). This includes many species of medical and agricultural significance, such as the facultative intracellular pathogens *Bartonella* and Brucella, the nitrogen-fixing plant symbiont *Sinorhizobium*, and the causative agent of crown gall disease, Agrobacterium tumefaciens (4, 5). In A. tumefaciens, Brucella abortus, and Sinorhizobium meliloti, unipolar growth is responsible for elongation of these rod-shaped species (1). Furthermore, differential subcellular muropeptide distributions have been observed in A. tumefaciens sacculi (6); however, the mechanisms that underlie polar PG biosynthesis remain poorly understood.

PG biosynthesis is an essential process that allows bacteria to grow and divide, faithfully reproducing their characteristic cell shape (7). During cell wall synthesis, the precursor disaccharide-pentapeptide subunit is synthesized in the cytoplasm, flipped across the membrane by MurJ, and cross-linked into the existing PG sacculus. PG assembly requires different classes of synthesis enzymes, including the penicillin-binding proteins (PBPs), which can be further divided into two classes. Class A PBPs are bifunctional enzymes that catalyze β-1,4 linkages between the *N*-acetylglucosamine (NAG) and *N*-acetylmuramic acid (NAM) sugars in a process called transglycosylation and also synthesize cross-links between peptides in a process known as transpeptidation (8). Transpeptidation by PBPs results in a covalent cross-link between the carboxyl group of the d-alanine (d-ala) at the fourth position of the donor strand and the amino group of the meso-diaminopimelic acid (mDAP) at the third position on the acceptor strand, resulting in a 4-3 (dd) cross-link, where the numbers correspond to the position of the cross-linked amino acid. The class B PBPs are monofunctional enzymes that have transpeptidase (TPase) activity (9). The shape, elongation, division, sporulation (SEDS) family proteins, RodA and FtsW, also possess glycosyltransferase (GTase) activity (10, 11). Current models of cell wall assembly, primarily derived from studies in E. coli and B. subtilis, maintain that SEDS proteins, in complex with their cognate class B PBP, are the primary drivers of cell wall synthesis and are required to sustain rod shape (12–14). Thus, RodA functions with PBP2 during elongation, while FtsW functions with PBP3 (encoded by *ftsI*) during cell division (14–16). The class A PBPs are currently thought to act independently from the Rod complex, functioning primarily in PG maintenance and repair (10, 17, 18).

The suite of cell wall synthesis enzymes encoded by the *Rhizobiales* is distinctly different from other bacterial orders. For example, the elongation-specific Rod complex of proteins, including PBP2, RodA, and MreBCD, are absent (1, 19), suggesting that RodA-PBP2 are not the primary drivers of elongation. In addition, the genomes of *Rhizobiales* encode a large number of ld-transpeptidase (LDT) enzymes compared to most laterally growing species. LDTs are a class of cell wall synthesizing enzymes, which carry out transpeptidation reactions that result in a covalent cross-link between the mDAP at the third position of the donor strand and the mDAP at the third position of the acceptor strand, resulting in a 3-3 (ld) cross-link (20). The cell wall of A. tumefaciens contains a high proportion (30%) of 3-3 cross-links (1), in contrast to laterally growing rod-shaped bacteria, where only 1 to 5% of the cell wall is cross-linked by LDTs (21). This suggests that LDT enzymes may play an important role during polar growth (1, 22). Overall, these observations suggest that *Rhizobiales* use a noncanonical mechanism for polar elongation.

Using a combination of microscopy, fluorescent amino acid and dipeptide probes, and biochemical and genetic analyses, we have characterized the function of the six high-molecular-weight PBPs encoded by A. tumefaciens and have identified the major cell wall synthesis enzymes required for polar growth. We show that, unlike the proposed auxiliary function of PBP1a in other rod-shaped bacteria, in A. tumefaciens, PBP1a is an essential enzyme required for polar PG expansion, with depletion of PBP1a resulting in a loss of proper rod shape. Using newly developed fluorescent d-amino acid dipeptide (FDAAD) probes, we show that PBP1a is the enzyme primarily responsible for inserting nascent PG at the pole. Additionally, PBP1a depletion leads to a modified PG composition, including an increase in LDT linkages. Collectively, this suggests that the mechanism of polar growth in the *Rhizobiales* has evolved through the expansion, diversification, and altered regulation of the core cell wall synthesis machinery. We confirmed the essentiality of PBP1a in the closely related bacterium *Sinorhizobium*, suggesting that the mechanisms underlying polar growth in the *Rhizobiales* are well conserved. Finally, we have identified the β-lactam faropenem as a specific inhibitor of polar growth in *Agrobacterium*, *Sinorhizobium*, and Brucella, indicating that the targets of faropenem are conserved among the *Rhizobiales*. These findings broaden our understanding of the role of PG synthesis enzymes that contribute to polar growth and will inform strategies aimed at developing novel therapeutics that target the cell wall of polar-growing bacteria in the *Rhizobiales* (23, 24).

## RESULTS

### The cell wall synthase PBP1a is essential for polar growth.

**FIG 1:**
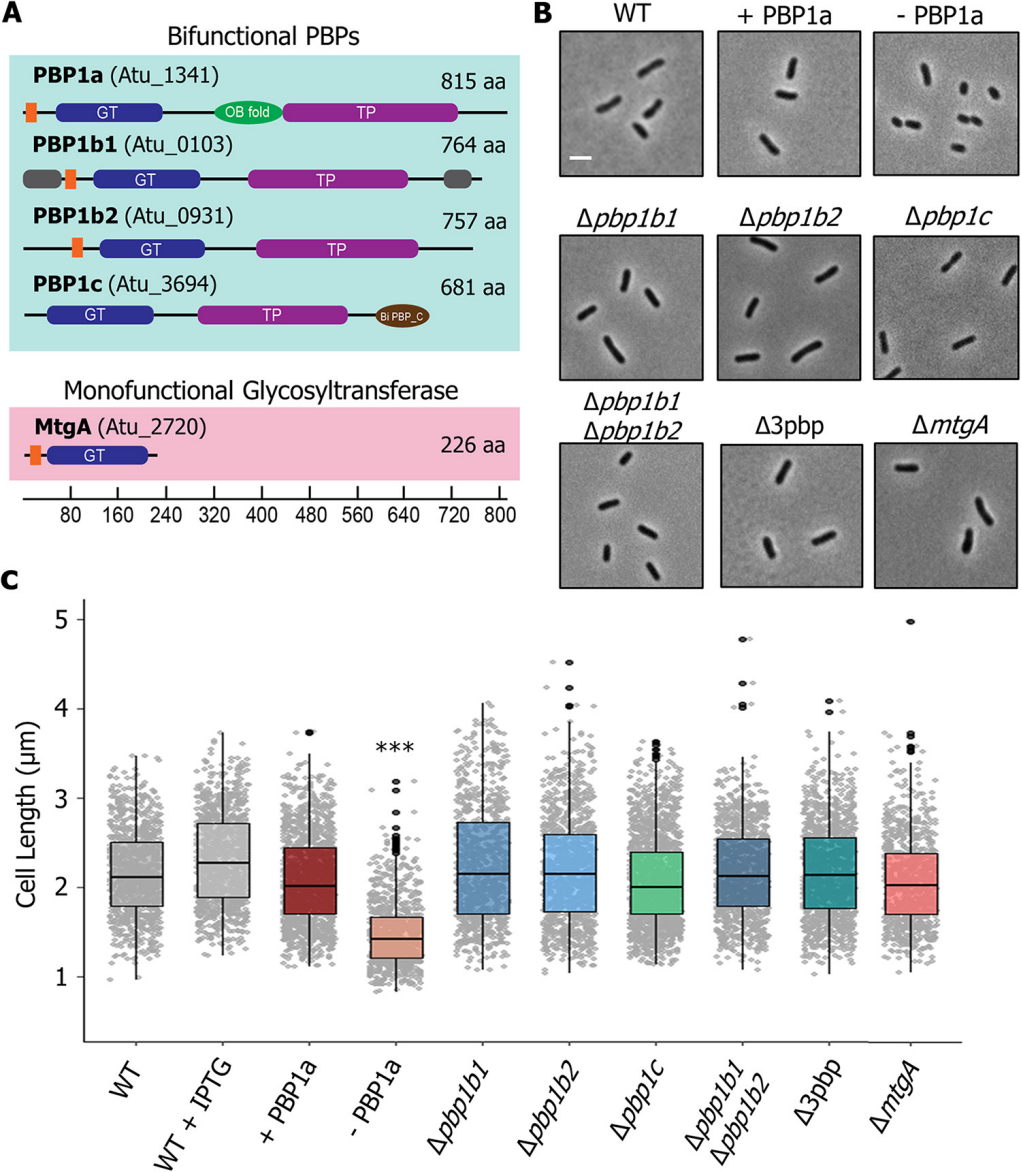
Functional characterization of PG synthases in A. tumefaciens reveals PBP1a is the major synthase for elongation. (A) Domain structure of the putative PG biosynthesis enzymes showing the transmembrane (orange), glycosyltransferase (GT, PF00912), transpeptidase (TP, PF00905), OB-like (PF17092), and biPBP_C (PF06832) domains. The regions of intrinsic disorder (gray) as predicted by MoBiDB are also shown (74). The scale indicates the length in amino acids (aa). The corresponding locus tags (Atu numbers) are listed in parentheses beside each gene name. (B) Phase microscopy images showing the phenotypes of PG synthase mutants. Each strain was grown to exponential phase, spotted on an ATGN agar pad [ATGN is a minimal medium with glucose and (NH_4_)_2_SO_4_], and imaged by phase microscopy. Scale bar = 2 μm. (C) Cell length distributions of PG synthase mutants. The indicated strains were grown as described for panel B and subjected to cell length measurements using MicrobeJ (73). The data are represented as box and whisker plots in the style of Tukey (75), which visualize five summary statistics (the center line is the median, the two hinges, corresponding to the first and third quartiles [the 25th and 75th percentiles], and the two whiskers [representing the smallest and largest values no further than 1.5 times the interquartile range]), with black circles indicating regions where outlying data points are observed. All data points are plotted individually in gray. The PBP1a depletion strain grown in the presence of IPTG is referred to as + PBP1a, and the depletion strain grown in the absence of IPTG is referred to as – PBP1a. Distributions of cells significantly different from wild type (WT) are indicated (***, one-way ANOVA with Bonferroni correction, *P* > 2 · 10^16^). *n* = >800 cells per strain.

**FIG 2:**
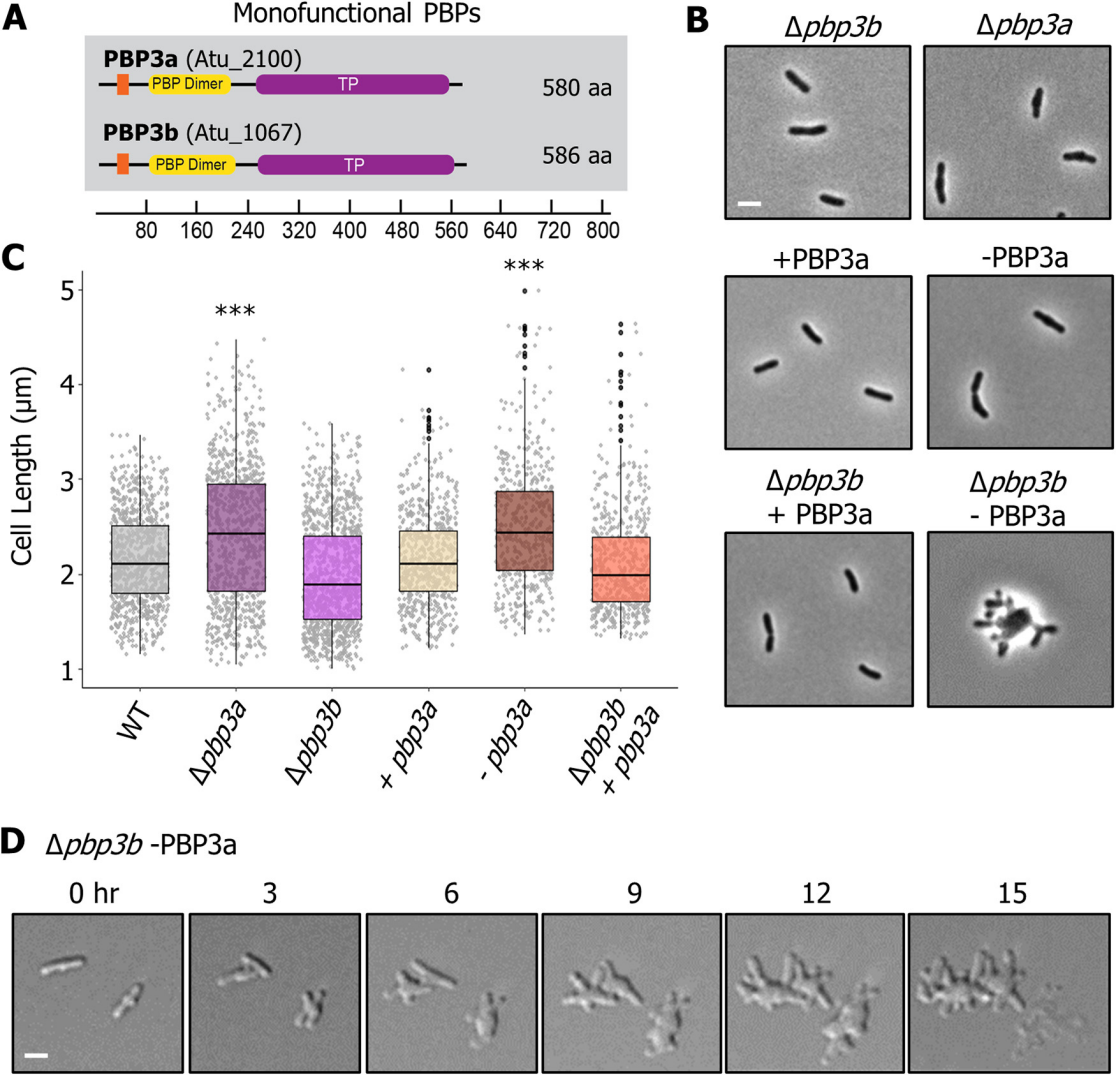
Functional characterization of monofunctional synthases PBP3a and PBP3b reveals they contribute to cell division. (A) Domain structure of the putative PG biosynthesis enzymes showing the transmembrane (orange), transpeptidase (TP, purple, PF00905), and PBP dimer (yellow, PF03717) domains. The scale indicates the length in amino acids (aa). The corresponding locus tags (Atu numbers) numbers are listed in parentheses beside each gene name. (B) Phase microscopy images showing the phenotypes of PG synthase mutants. Each strain was grown to exponential phase, spotted on an ATGN agar pad, and imaged by phase microscopy. Scale bar = 2 μm. (C) Cell length distributions of PG synthase mutants. The indicated strains were grown to exponential phase, spotted on an agar pad, imaged by phase microscopy, and subjected to cell length measurements using MicrobeJ (73). The data are shown as box and whisker plots in the style of Tukey (75) as described in the legend for Fig. 1. Distributions of cells significantly different from the wild type (WT) are indicated (***, one-way ANOVA with Bonferroni correction, *P* < 2 · 10^16^). *n* = >500 per strain. (D) DIC image series of the Δ*pbp3b* -PBP3a strain. Cells were predepleted of IPTG for 4 h in liquid culture before spotting onto a 1% ATGN agar pad. Time in hours (h).

To begin probing the molecular determinant(s) of polar growth in A. tumefaciens, we sought to generate deletions of the predicted PG synthase enzymes. Although A. tumefaciens lacks homologs to the predicted cell elongation synthases RodA and PBP2, the genome encodes four bifunctional class A PBPs (PBP1a, PBP1b1, PBP1b2, and PBP1c), two monofunctional class B PBPs (PBP3a and PBP3b), and one monofunctional GTase, MtgA (Fig. 1A and 2A). To determine which of these enzymes provide the primary GTase activity in the absence of a RodA homolog, we made in-frame deletions of those genes encoding predicted GTase enzymes to further explore their contribution during cell growth or division.

Consistent with observations of citrine-PBP1 at the growth pole (19) and a saturating transposon mutagenesis screen (25), it was not possible to obtain a PBP1a deletion, suggesting that this enzyme may have an essential function during polar growth. We therefore constructed a PBP1a depletion strain by introducing a copy of the PBP1a-encoding gene, under an IPTG (isopropyl-β-d-thiogalactopyranoside)-inducible promoter, at a heterologous site on the chromosome, and subsequently succeeded in creating an in-frame deletion of the native gene encoding PBP1a in the presence of the IPTG inducer (26). The PBP1a depletion strain grown in the presence of IPTG is referred to as + PBP1a, and the depletion strain grown in the absence of IPTG is referred to as – PBP1a. We confirmed depletion of PBP1a in the absence of IPTG using Bocillin-FL, a fluorescent penicillin derivative. Two bands were observed that could correspond to the predicted molecular weight of PBP1a (∼88 kDa), but only the second band was absent in PBP1a-depleted cells and likely represents PBP1a protein (see Fig. S1A). Strikingly, cells depleted of PBP1a for 16 h lost their rod shape, becoming shorter (Fig. 1A and B) and wider (see Fig. S1B) and had a severe viability defect, as measured by spotting serial dilutions, compared to the same strain when PBP1a is induced (see Fig. S1C). The addition of IPTG to wild-type A. tumefaciens led to a slight increase in the median cell length (Fig. 1B and C) compared to the wild type alone but had no effect on cell viability or rod shape (see Fig. S1B and C).

10.1128/mBio.02346-21.2 FIG S1 PBP mutants impact cell viability. (A) Bocillin labeling of cell membrane preparations of the wild-type (WT) and the PBP1a depletion strain. The PBP1a depletion strain was grown in the presence (+ PBP1a) or absence of 1 mM IPTG for 16 h (– PBP1a). The band corresponding to PBP1a protein is not detected after 16 h of depletion. (B) Cell width distributions of wild-type cells compared to the PBP1a depletion strain grown with or without IPTG for 16 h. The indicated strains were grown as described in Fig. 1 and subjected to cell width measurements using MicrobeJ (73). The data are shown as box and whisker plots in the style of Tukey (75). Distributions of cells significantly different from the WT are indicated (***, one-way ANOVA with Bonferroni correction, *P* < 2 · 10^16^). *n* = >800 per strain. (C) Spot assay for cell viability of the PBP1a depletion strain. Images are representative of three independent biological replicates. Dilutions are indicated above each spot, and depletion strains are indicated in the boxes. (D) Spot assays for cell viability of bifunctional PBP deletion strains. Dilutions are indicated above each spot, and images are representative of three independent biological replicates. (E) Bocillin labelling of WT cell membrane preparation, which is a representative image of two independent experiments. Each PBP is labeled according to its predicted molecular weight. (F) Spot assays for cell viability of monofunctional PBP deletion and depletion strains. Dilutions are indicated above each spot, and depletion strains are indicated in the boxes. Images are representative of three independent biological replicates. Download FIG S1, TIF file, 1.1 MB. Copyright © 2021 Williams et al. 2021 Williams et al. https://creativecommons.org/licenses/by/4.0/ This content is distributed under the terms of the Creative Commons Attribution 4.0 International license.

Of the remaining bifunctional PBPs, single deletions of the genes encoding PBP1b1, PBP1b2, and PBP1c had no effect on cell length (Fig. 1B and C) or cell viability (see Fig. S1D). Similarly, a double mutant of PBP1b1 and PBP1b2 or a triple mutant of PBP1b1, PBP1b2, and PBP1c (referred to as Δ3pbp) had no obvious mutant phenotype with respect to cell length (Fig. 1B and C) or cell viability (see Fig. S1D). Thus, despite all of the predicted bifunctional PBPs being produced during exponential growth of A. tumefaciens (see Fig. S1E), our data indicate that only PBP1a makes a major contribution to cell growth under standard laboratory conditions. Similarly, deletion of the monofunctional GTase encoding *mtgA* produced cells of normal length (Fig. 1B and C). The lack of a readily observed phenotype in the Δ*mtgA* strain is consistent with findings in E. coli and Hyphomonas neptunium (27–29). Together, these data suggest that the bifunctional enzyme PBP1a, which likely has both GTase and TPase activities, fulfils the role of RodA and PBP2 as the primary PG synthase required for polar elongation in A. tumefaciens.

### Class B synthases PBP3a and PBP3b are required for cell division.

Incorporation of PG at the septum prior to cell division typically requires synthesis enzymes that are distinct from the cell elongation machinery. Although A. tumefaciens lacks the cognate SEDS-PBP pair that is typically required for elongation, the SEDS protein FtsW and PBP3, which are required for cell division, are conserved. While most bacteria possess a single, essential *ftsI* gene that encodes PBP3, some *Rhizobiales*, including A. tumefaciens, encode two FtsI homologs (PBP3a and PBP3b) (Fig. 2A). *pbp3a* resides in the *mra* operon of cell division and cell envelope biogenesis genes, similar to most *ftsI*-encoding homologs (30), while PBP3b is encoded as a monocistronic gene elsewhere in the genome. This raises the possibility that the second monofunctional transpeptidase (PBP3b) may serve the role of a PBP2 homolog that functions in polar elongation. Thus, we sought to determine if either of the two class B PBP homologs were required for cell division or polar elongation in A. tumefaciens. Saturating transposon mutagenesis in LB medium indicated that PBP3a was likely essential (25); however, it was possible to make a deletion of *pbp3a* when cells were grown in minimal medium. These observations suggest that the PBP3a-encoding gene was conditionally essential.

In minimal medium, deletion of *pbp3a* caused a severe cell viability defect (see Fig. S1F). In addition, cells were longer (Fig. 2B and C) and were frequently observed to adopt branching or bulging morphologies; these features are a hallmark of cell division defects in A. tumefaciens (31, 32). In contrast, deleting *pbp3b* had no effect on cell viability (see Fig. S1F) or cell length (Fig. 2B and C). Together, these results indicate that PBP3a is the major class B PBP contributing to cell division in A. tumefaciens. Since deletion of the gene encoding PBP3a did not fully inhibit cell division, we hypothesized that PBP3b may be able to partially compensate for the loss of PBP3a. To address this possibility, we first created a PBP3a depletion strain; when this strain was grown in the absence of IPTG for 24 h, it phenocopied the cell viability and cell length defects of the *pbp3a* deletion mutant (Fig. 2B and C). We then depleted PBP3a in a Δ*pbp3b* mutant background and found that cells not only failed to divide, but also swelled at the midcell before lysing (Fig. 2C and D and Movie S1), indicating that PBP3a and PBP3b both contribute to septal PG biosynthesis during cell division. The phenotype observed in the absence of both PBP3a and PBP3b was remarkably similar to the phenotype observed during depletion of FtsW in A. tumefaciens (31). Consistent with current models of cell division, we hypothesize that FtsW provides the GTase activity, while PBP3a and PBP3b provide the dd-transpeptidase (TPase) activity necessary for proper septal PG biosynthesis during cell division in A. tumefaciens. In all, our findings support a model in which PBP3a can sustain proper cell division in the absence of PBP3b and that, while PBP3b contributes to septal PG synthesis, it cannot fully compensate for the loss of PBP3a. Thus, both class B PBPs contribute primarily to septal PG biosynthesis.

10.1128/mBio.02346-21.8 MOVIE S1 Cells lacking both PBP3a and PBP3b are blocked for cell division. DIC image series of the PBP3b deletion PBP3a depletion strain. Cells were predepleted of IPTG for 4 h before spotting onto a 1% ATGN agar pad. Images were acquired every 10 min, and the movie is played at 8 frames per second for a total of 112 frames Download Movie S1, AVI file, 8.8 MB. Copyright © 2021 Williams et al. 2021 Williams et al. https://creativecommons.org/licenses/by/4.0/ This content is distributed under the terms of the Creative Commons Attribution 4.0 International license.

### Development of fluorescent cell wall probes to monitor PBP activity.

**FIG 3:**
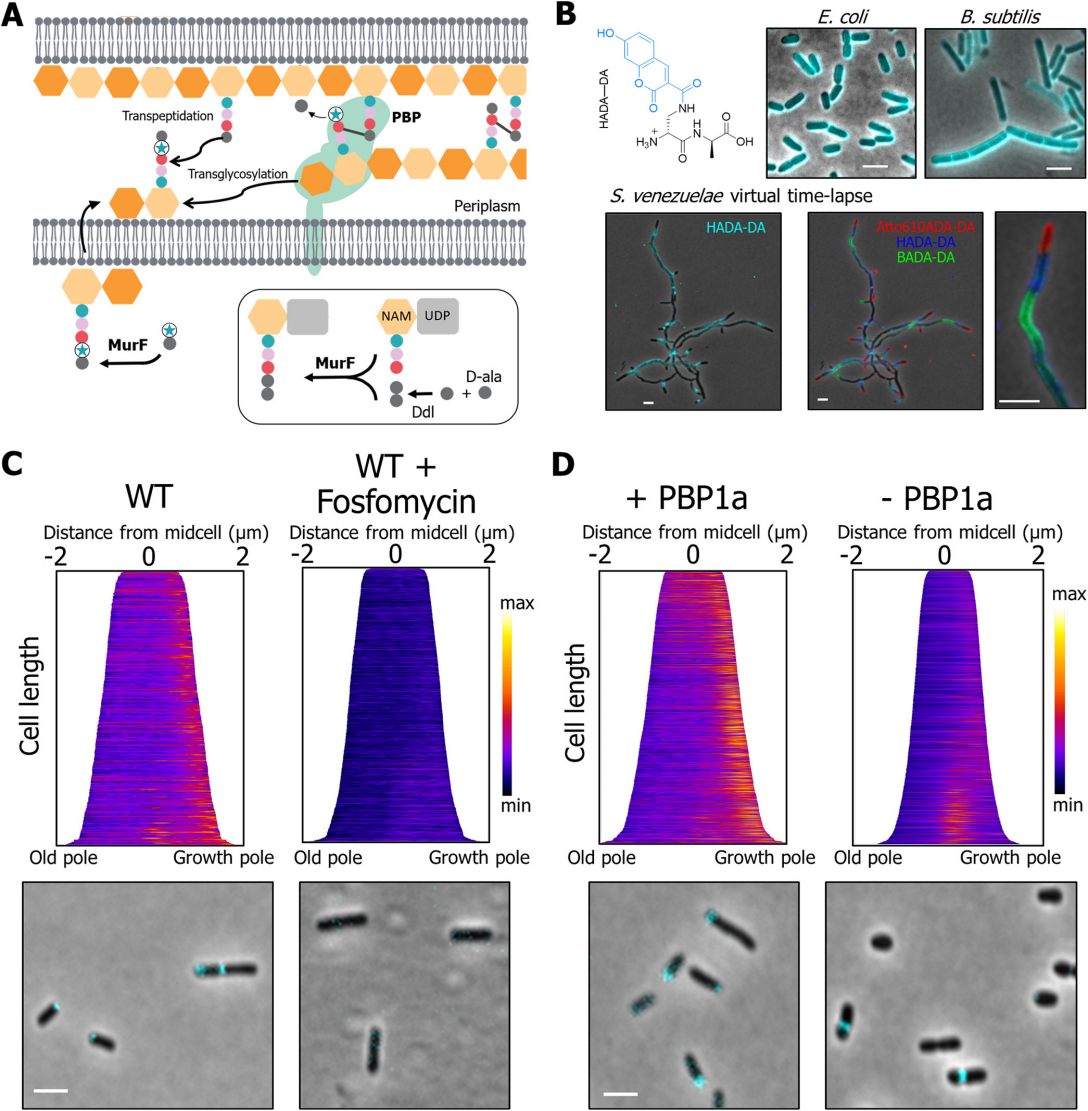
Fluorescent d-amino acid dipeptide (FDAAD) labeling is absent from the growth pole in the PBP1 depletion. (A) Schematic of the incorporation pathway of fluorescent d-amino acid dipeptides (FDAADs), which are incorporated into the muropeptide precursor molecule in the cytoplasm by MurF ligase. The modified muropeptide precursor is flipped across the membrane, and the activity of a bifunctional PBP cross-links the new PG monomer into the existing PG sacculus. (B) (Top row) Structure of the FDAAD HADA–DA and merged phase and fluorescent channels of labeling patterns of HADA–DA in E. coli and B. subtilis. (Bottom row) Short pulse-labeling of *S. venezuelae* sequentially labeled first with BADA–DA, followed by HADA–DA and Atto610DA–DA. (C) Demographs depict incorporation of FDAADs at a population level of the wild type (WT) or the WT treated for 1 h with 30 mM fosfomycin. Median profiles of the fluorescence channel are stacked and ordered by cell length; *n* = >500 per strain. Merged phase and fluorescent channels of cells with representative HADA–DA labeling are shown below each demograph. Scale bars = 2 μm. (D) Demographs depict incorporation of FDAADs in the PBP1a depletion strain grown in the presence or absence of IPTG. Strains are labeled as + PBP1a and – PBP1a, respectively. Merged phase and fluorescent channels of cells with representative polar and septum labeling of HADA–DA are shown below each demograph. Scale bars = 2 μm. *n* = >1,000 per strain.

Traditional fluorescent d-amino acid (FDAA) probes are an exceptionally useful tool for investigating the patterning of cell wall synthesis in diverse microbes (33). However, FDAAs report on the activity of extracellular/periplasmic dd and ld transpeptidases and as a result can be incorporated into the PG in a growth-independent mechanism (6, 20). Here, we have developed fluorescent d-amino acid dipeptide (FDAAD) probes to observe nascent sites of PG cross-linking in living cells, thus eliminating the need for click chemistry that is required when using traditional d-amino acid dipeptide (DAAD) probes (6, 34). DAADs are incorporated into the cell wall precursors by the cytoplasmic MurF ligase, and probe incorporation reports specifically on nascent PG synthesis (34–36). The resulting lipid II-linked modified precursor is most likely covalently cross-linked to an existing glycan strand through the activity of bifunctional PBPs (Fig. 3A). The FDAAD probe HCC-amino-d-alanine-d-alanine (HADA–DA) successfully labeled the PG of several bacteria, including B. subtilis, E. coli, Streptomyces venezuelae, and A. tumefaciens (Fig. 3B and C), and we demonstrated and evaluated PG labeling with four additional FDAADs of different sizes and molecular weights in diverse species (see Fig. S2A to D). In addition, *S. venezuelae*, a polar-growing *Actinobacteria*, was short pulsed with three different FDAADs to illustrate that these probes report on the newest PG synthesis activity (Fig. 3B), similar to FDAAs (37, 38). Given their cytoplasmic mechanism of incorporation, complementary to FDAAs (which are incorporated by transpeptidases periplasmically) (6), these probes will be particularly useful to distinguish between growth-dependent and growth-independent PG cross-linking in species with a higher proportion of extracellular LDT activity, such as polar-growing bacterial species.

10.1128/mBio.02346-21.3 FIG S2 FDAAD probes show distinct labeling patterns in different bacteria. (A) Structures of the fluorescent d-amino acid dipeptides (FDAADs) investigated in this work. (B) Table indicating the labelling pattern of different fluorescent d-amino acid dipeptides (FDAADs) in different bacterial species. BADA–DA is a brighter fluorescent alternative to NADA–DA in most cases. +, Significant labeling (signal-to-background, >1); NT, not tested; *, a quantitative comparison between minimal medium, e.g., M9, and rich medium, e.g., LB, where minimal medium showed stronger labeling; **, patchy or peculiar labeling patterns different from FDAA labeling; ***, where a quantitative comparison between DA-NADA and NADA–DA is done and labeling shows weaker DA-NADA labeling, likely due to the loss of the fifth position NADA upon cross-linking by PBPs. (C) Merged phase and fluorescent images of representative cells comparing labeling between NADA–DA and DA-NADA in the species listed. (D) Merged phase and fluorescent images of representative cells comparing labeling between Atto610ADA–DA and BADA–DA. All scale bars are 2 μm. (E) Comparison of the labelling patterns of different fluorescent cell wall probes in the Δ*pbp3* strain. Demographs depict incorporation of either FDAADs or FDAAs at a population level. Median profiles of the fluorescence channel of more than 1,200 cells per strain are stacked and ordered by cell length. Merged phase and fluorescent channels of cells with representative polar and septal labeling of fluorescent probes are shown beside each demograph. Scale bars = 2 μm. (F) The demograph depicts incorporation of HADA after a 1-h incubation of WT cells with 30 mM fosfomycin. More than 1,200 cells are stacked and ordered by cell length. Download FIG S2, TIF file, 2.8 MB. Copyright © 2021 Williams et al. 2021 Williams et al. https://creativecommons.org/licenses/by/4.0/ This content is distributed under the terms of the Creative Commons Attribution 4.0 International license.

### PBP1a is the major synthase incorporating PG at the pole.

A. tumefaciens was labeled for ∼5% of the cell cycle with HADA–DA. As expected, cells exhibit labeling at the growth pole during elongation and at the septum in cells undergoing cell division (Fig. 3C). This pole-to-septum labeling pattern is consistent with other cell wall labeling methods, including fluorescent d-amino acids (FDAAs) (39) and d-cysteine labeling (1). Similar to wild-type cells, a pole-to-septum labeling pattern was observed in the Δ3pbp mutant, consistent with a limited role for these class A PBPs in polar growth under the conditions tested (see Fig. S2E). To confirm cytoplasmic incorporation of the FDAADs, we treated wild-type cells for 1 h with the MurA inhibitor fosfomycin, which blocks the first step in cell wall precursor synthesis (40, 41). As expected, based on the mechanisms of incorporation for other DAAD probes (6, 34–36), fosfomycin treatment blocked both polar and midcell labeling of HADA–DA (Fig. 3C), while some periplasmic HADA labeling was still evident (See Fig. S2F).

In stark contrast to wild-type (Fig. 3C) or PBP1a-replete cells (Fig. 3D), pole-specific labeling with HADA–DA was almost completely absent from cells depleted of PBP1a for 20 h (Fig. 3D). Similar results were observed with NADA–DA and BADA–DA (see Fig. S3A and B). Consistent with these observations, we tracked the growth of PBP1a-depleted cells for seven generations using a microfluidic device and found that the reduction in cell length occurred first for the new pole daughter cell and likely resulted from the loss of polar PG insertion by PBP1a (see Fig. S4A, and Movie S2). For the first 3 generations, the growth rate is maintained as the daughter cells get shorter; however, the growth rate declines dramatically in later generations (see Fig. S4B), suggesting that loss of PBP1a is not sustainable. We thus concluded that PBP1a is the major PG synthase required for polar PG incorporation. These findings are consistent with the observation of Bocillin-FL labeling and citrine-PBP1a at the growth pole (19). Notably, PBP1a-depleted cells have more robust FDAAD labeling at the septum (Fig. 3D), suggesting that additional glycosyltransferase enzymes remain active at the site of cell division.

10.1128/mBio.02346-21.4 FIG S3 NADA–DA and BADA–DA display similar labelling patterns and are absent from the pole in the PBP1a depletion. (A) Demographs depict incorporation of NADA–DA at a population level. Median profiles of the fluorescence channel of more than 400 cells per strain are stacked and ordered by cell length. Merged phase and fluorescent channels of cells with representative polar and septal labeling of dipeptides are shown below each demograph. (B) Demographs depict incorporation of BADA–DA at a population level. Median profiles of the fluorescence channel of more than 400 cells per strain are stacked and ordered by cell length. Merged phase and fluorescent channels of cells with representative polar and septal labeling of dipeptides are shown below each demograph. Scale bar = 2 μm. Download FIG S3, TIF file, 2.3 MB. Copyright © 2021 Williams et al. 2021 Williams et al. https://creativecommons.org/licenses/by/4.0/ This content is distributed under the terms of the Creative Commons Attribution 4.0 International license.

10.1128/mBio.02346-21.5 FIG S4 Impacts of the PBP1a depletion accumulate over multiple generations. (A) Shown are cell length distributions of the PBP1a depletion strain grown in a microfluidic device. A timeline of the experiment is provided. Cells were induced with 1 mM IPTG for 4 h before washing to deplete the cells of PBP1a for the remainder of the experiment. Twelve cells were followed for seven generations, corresponding to 24 h of growth in the device, and the cell length of the two daughter cells was measured one frame after the cell divided (D1 and D2 for daughter cell 1 and daughter cell 2). D1 was always designated as the cell that remained attached to the microfluidic device, or the old pole daughter cell. Rarely, if D2 (the new pole daughter cell) washed away before it could be measured, the cell length was calculated by subtracting the length of D1 from the mother cell length just before it divided. Cell length measurements were collected using MicrobeJ (73). The data are represented as box and whisker plots in the style of Tukey (75). Phase images of one representative cell just before cell division and just after cell division from four different generations (gen) corresponding to predepletion and early, mid-, and late depletion timepoints. Time is indicated in minutes. (B) The growth rate in hours (h) of the 12 cells in panel A was calculated by taking the inverse of the time between cell divisions. (C) Comparison of the labelling pattern after a long pulse with FDAAs and FDAADs. The demographs depict incorporation of either HADA or HADA–DA at a population level. Median profiles of the fluorescence channel of 95 cells for HADA and 190 cells for HADA–DA are stacked and ordered by cell length. The scale bar for the HADA demograph represents intensity and ranges from 900 to 4,800 arbitrary units (a.u.). The scale bar for the HADA–DA demograph ranges from 100 to 900 a.u. Merged phase and fluorescent channels of cells with representative polar and septal labeling of dipeptides are shown beside each demograph. Brackets point to the sidewalls of the new pole. Below the demographs are images of several representative cells; the fluorescence and phase are shown separately. Scale bar = 2 μm. Download FIG S4, TIF file, 2.1 MB. Copyright © 2021 Williams et al. 2021 Williams et al. https://creativecommons.org/licenses/by/4.0/ This content is distributed under the terms of the Creative Commons Attribution 4.0 International license.

10.1128/mBio.02346-21.9 MOVIE S2 PBP1a-depleted cells get successively smaller and rounder over multiple generations in a microfluidic device. Phase image series of the PBP1a depletion strain growing in ATGN minimal medium in a microfluidic device. Cell were grown in the presence of IPTG to induce PBP1a expression for 4 h, washed of IPTG, and grown without inducer for the remainder of the video. Images were acquired every 10 min, and the movie is played at 10 frames per second for a total of 123 frames. Download Movie S2, AVI file, 0.3 MB. Copyright © 2021 Williams et al. 2021 Williams et al. https://creativecommons.org/licenses/by/4.0/ This content is distributed under the terms of the Creative Commons Attribution 4.0 International license.

### ld-transpeptidases contribute to PG modification at the growth pole and along the sidewall of the growth pole compartment.

**FIG 4:**
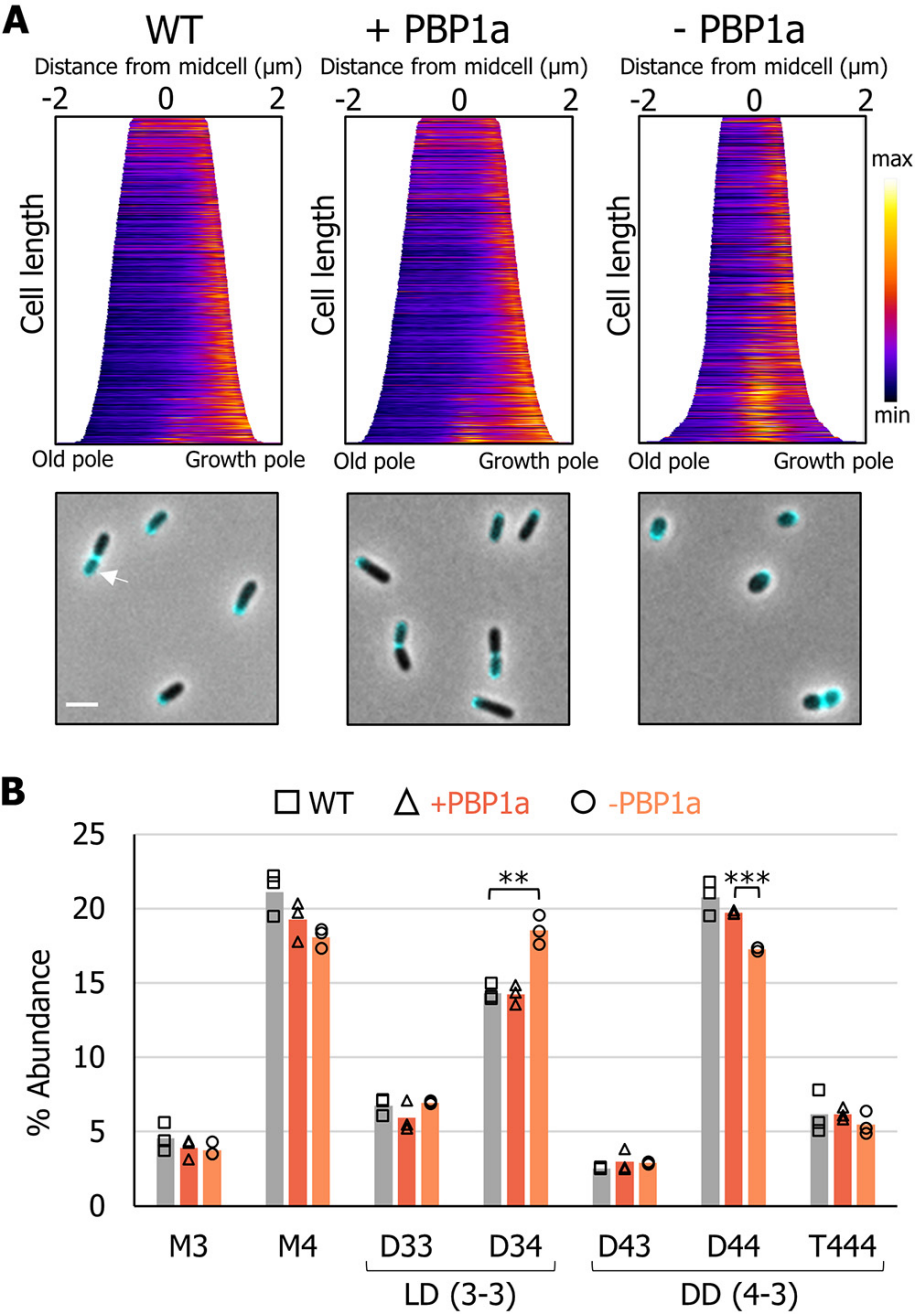
Fluorescent d-amino acid (FDAA) and PG composition analysis illustrates a role for LDTs in polar growth. (A) Demographs of the wild-type (WT) and the PBP1a depletion strain grown in the presence or absence of IIPTG as indicated by + PBP1a and – PBP1a, respectively. Demographs depict incorporation of FDAA at a population level. Median profiles of the fluorescence channel are stacked and ordered by cell length *n* = >600 per strain. Shown below each demograph are merged phase and fluorescent channels of cells with representative polar and septum labeling by FDAAs. Scale bar = 2 μm. (B) Bar graphs depicting the average abundance of muropeptides obtained by UPLC analysis from the wild-type and the PBP1a depletion strain grown in the presence or absence of IPTG for 16 h. Major muropeptides are labeled M, monomers; D, dimers; and T, trimers. Numbers indicate the length of the muropeptide stems and the position of cross-links in dimers and trimers. The data shown are averages taken from analyses of three independent biological samples. Samples that are statistically significant are indicated (one-way ANOVA with Tukey’s multiple-comparison test; **, *P* < 0.005; ***, *P* < 0.0005). The *P* value between + PBP1a and – PBP1a for D34 was 0.0588 and between the WT and – PBP1a for D44 was 0.072.

In contrast to FDAAD incorporation, which is linked to the PG precursors in the cytoplasm, the more conventional fluorescent d-amino acid (FDAA) labeling occurs in the periplasm through either dd-transpeptidase reactions carried out by PBPs or ld-transpeptidase reactions carried out by LDTs (39, 42). As expected, wild-type and Δ3pbp cells labeled with a short pulse of the FDAA probe HADA show the characteristic pole-to-septum labeling pattern that is typical of A. tumefaciens growth (Fig. 4A and Fig. S2E). Notably, FDAADs label discrete regions at the pole and midcell, while FDAA labeling is more prominent in the sidewalls, particularly of the new pole compartment prior to cell division, while the old pole remains unlabeled. (Fig. 4A, white arrow). To further explore this observation, we labeled A. tumefaciens for 60 min with either HADA or HADA–DA and compared the labeling patterns (see Fig. S4C). After a 60-min incubation, HADA–DA labeling was primarily found in distinct regions at the pole and midcell, with little sidewall labeling, similar to a short pulse and consistent with areas of new PG synthesis. In contrast, the HADA labeling was much brighter and fully labeled the sidewalls. HADA labeling was particularly enriched along the sidewalls of the new-pole compartment. Our observations during short- and long-pulse-labeling experiments suggest that, in addition to growth pole and midcell labeling, LDTs contribute to spatially distinct cross-linking of the cell wall, particularly along the sidewalls of the new-pole daughter cell.

We next sought to test whether FDAA labeling at the growth pole was absent in the PBP1a depletion strain. In contrast to the absence of polar incorporation that was seen following FDAAD labeling, cells depleted of PBP1a labeled robustly at the growth pole with HADA (Fig. 4A). Since PBP-mediated incorporation of FDAADs is absent from the growth pole in the PBP1a depletion (Fig. 3D and Fig. S3A and B), the major enzymes incorporating HADA at the pole are most likely LDTs, consistent with recent findings for incorporation of FDAAs in E. coli (42). Additionally, the activity of LDTs has been shown to be functionally linked to the activity of class A PBPs (42, 43).

### Depletion of PBP1a or loss of PBP1b2 leads to increased levels of ld-cross-links in PG.

Since depletion of PBP1a led to dramatic reduction in the polar incorporation of FDAADs, while maintaining polar FDAA incorporation, we hypothesized that a decrease in the activity of PBPs relative to LDTs may lead to altered PG composition. We grew the PBP1a depletion strain in the presence or absence of IPTG for 16 h, collected the cell wall fraction, and analyzed muropeptides by ultraperformance liquid chromatography (UPLC). The major muropeptides found in wild-type A. tumefaciens PG included monomeric (M), dimeric (D), and trimeric (T) muropeptides (Fig. 4B). Muropeptide dimers and trimers contain a dd (4-3) or ld (3-3) cross-link, depending on whether they were cross-linked by a PBP or LDT enzyme, respectively. While muropeptides are cross-linked in a specific (4-3 or 3-3) position, the peptide length of the donor and acceptor strands can vary. Each muropeptide is designated with an M, D, or T followed by two numbers that correspond to the length of the peptide stem of the donor and acceptor strands. Thus, both D33 and D34 contain ld-cross-links, whereas D43 and D44 have dd-cross-links. PG from PBP1a-depleted cells had significantly reduced levels of muropeptides with dd-cross-links, as seen by the ∼3% reduction in D44 abundance (Fig. 4B). As expected, this observation confirmed that depleting PBP1a leads to decreased dd‐transpeptidase activity. Depletion of PBP1a also resulted in an increase in muropeptides containing ld-cross-links, as indicated by the ∼4% increase in D34 abundance (Fig. 4B). A similar decrease in D44 abundance and increase in D34 abundance was also observed in the Δ*pbp1b2* (see Fig. S5A) but not in the single deletions of *pbp1b1*, *pbp1c*, *pbp3a*, or *pbp3b* (see Fig. S5B). Therefore, the compositional changes in the double *pbp1b1 pbp1b2* mutant strain can be attributed to the deletion of *pbp1b2* (see Fig. S5A). These results implicate PBP1b2 as an important dd-transpeptidase enzyme in A. tumefaciens and suggest that increased LDT activity may be a general response to decreased dd-transpeptidase levels and not necessarily a specific response to depletion of PBP1a. Furthermore, since we saw an increase in muropeptide D34 but not D33 (Fig. 4B), we hypothesize that decreased dd-transpeptidase levels activate only a subset of LDTs and that another group of LDTs may function along with PBP1a during polar growth, consistent with the observation of HADA labeling at the growth pole.

10.1128/mBio.02346-21.6 FIG S5 PBP mutants and antibiotic treatment alters peptidoglycan composition. (A) Bar graphs showing the average abundance of muropeptides obtained by UPLC analysis from the indicated strains. The data shown are averages taken from analyses of three independent biological samples. Significantly different samples are indicated (one-way ANOVA with Tukey’s multiple-comparison test; *, *P* < 0.05; **, *P* < 0.005). (B) Bar graphs showing the average abundance of muropeptides obtained by UPLC analysis from the indicated strains. The data shown are averages taken from analyses of three independent biological samples. The difference between samples was not significant (one-way ANOVA with Tukey’s multiple-comparison test). (c) Impact of antibiotic treatment on peptidoglycan composition of A. tumefaciens representative UPLC profiles showing the abundance of muropeptides obtained from the indicated strains. Cells were grown in the presence of dimethyl sulfoxide (DMSO) or 1.5 μg/ml faropenem for 6 h, corresponding to the onset of polar swelling induced by faropenem treatment before harvesting PG. Cells were grown in the presence of 1.5 μg/ml meropenem for 4 h, corresponding to the onset of midcell swelling induced by meropenem treatment before harvesting PG. Download FIG S5, TIF file, 0.7 MB. Copyright © 2021 Williams et al. 2021 Williams et al. https://creativecommons.org/licenses/by/4.0/ This content is distributed under the terms of the Creative Commons Attribution 4.0 International license.

### Faropenem treatment inhibits polar growth in the *Rhizobiales*.

**FIG 5:**
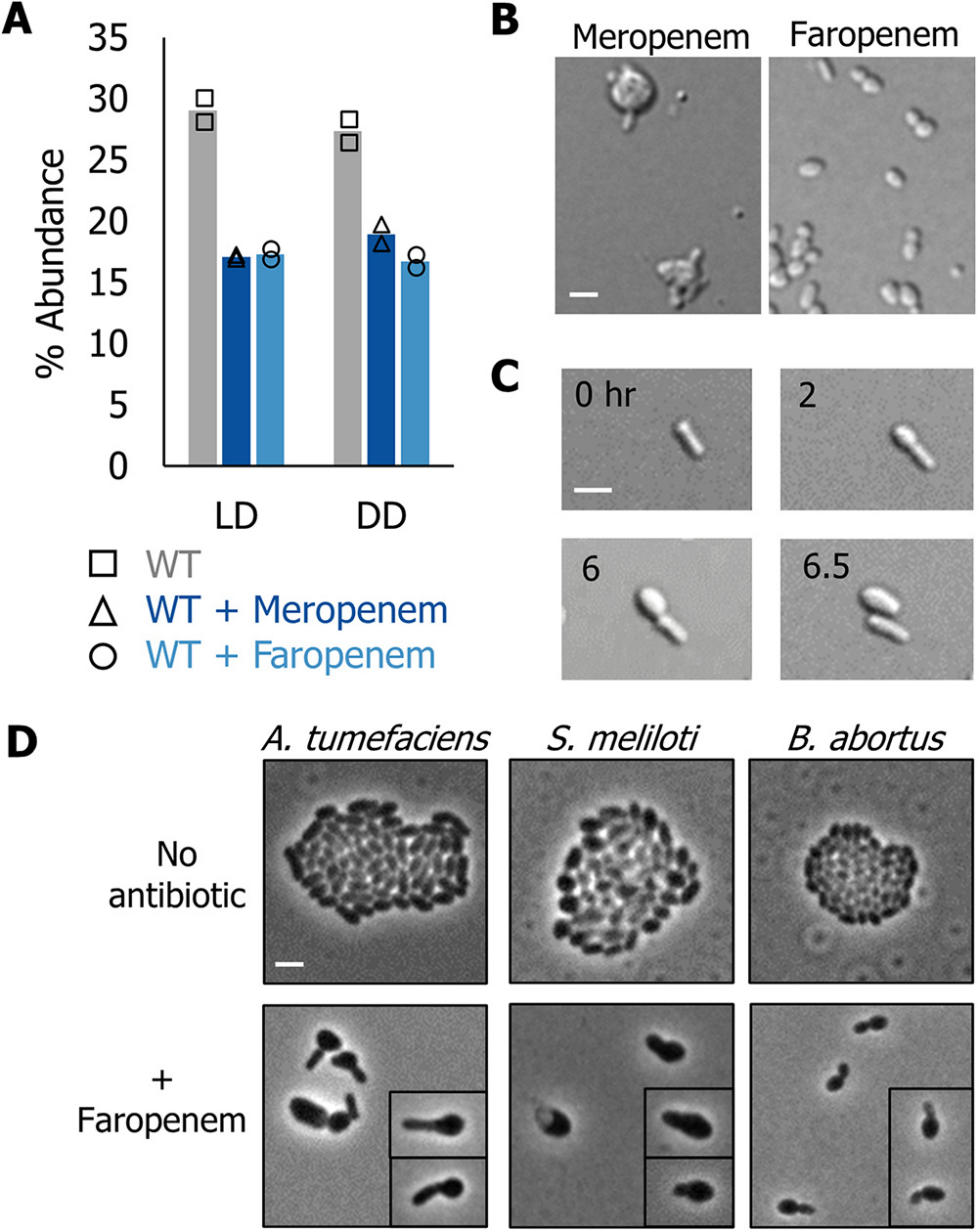
Phenotypic characterization of carbapenem and penem antibiotic treatments reveals loss of rod shape. (A) Abundance of total ld- and dd-cross-linkages in PG isolated from wild-type cells, wild-type cells grown in the presence of 1.5 μg/ml meropenem for 4 h, and wild-type cells (WT) grown in the presence of 1.5 μg/ml of faropenem for 6 h. Time points were chosen based on the onset of phenotypic changes. The data shown are the total abundance of the muropeptides containing ld- or dd-cross-links from analyses of two independent samples. (B) Representative images of wild-type cells grown in the presence of 1.5 μg/ml meropenem and faropenem. Cells were incubated with antibiotics for 24 h and then spotted on a 1% agarose pad and imaged using DIC microscopy. Scale bar = 2 μm (C) Time-lapse microscopy of wild-type cells spotted on a 1% ATGN agarose pad supplemented with 1.5 μg/ml of faropenem; images were acquired every 10 min. The indicated time in hours is shown. Scale bar = 2 μm. (D) Representative phase microscopy images of A. tumefaciens, S. meliloti, and B. abortus grown overnight in ATGN, tryptone-yeast (TY) extract, and Brucella broth, respectively, to an OD_600_ of 0.6 and spotted on a 1% ATGN agarose pad with or without 1.5 μg/ml faropenem. Cells were imaged after 16 h of growth. Scale bar = 2 μm.

Since species in the *Rhizobiales* have a large number of ld-transpeptidase enzymes (A. tumefaciens encodes 14 LDTs), deleting them all is a significant undertaking. β-Lactam antibiotics are one of the most widely used classes of antibiotics that target cell wall synthesis enzymes, and these have been primarily studied for their ability to target PBPs (44). A subclass of β-lactam antibiotics known as the carbapenems, including the penem antibiotic faropenem, have proven useful for probing the activity of LDTs in Mycobacterium (45, 46). Although the carbapenem and penem classes of antibiotics have yet to be characterized in A. tumefaciens, it is likely that the mode of action of these antibiotics is conserved based on the high functional similarity of cell wall enzymes across bacterial species. Therefore, to characterize the global contribution of LDT activity during A. tumefaciens growth, we investigated the effects of five carbapenem antibiotics and one penem antibiotic on cell growth and morphology. In A. tumefaciens, we find that treatment with meropenem or faropenem leads to an overall decrease in PG cross-linkage, including both ld- and dd-cross-links (Fig. 5A and Fig. S5C), suggesting that the targets of these drugs impact cell wall biosynthesis.

To better understand the impacts of these antibiotics, we observed morphological changes induced by drug treatment. Treatment with sub-minimum inhibitory concentrations (MIC) with any of five carbapenem antibiotics, meropenem, imipenem, doripenem, ertapenem, or tebipenem, for 24 h induced midcell swelling (Fig. 5B, and Fig. S6A), indicating that these carbapenem antibiotics targeted an enzyme(s) with a specific role at the septum during cell division. Interestingly, faropenem-treated cells became shorter and wider after a 24-h exposure (Fig. 5A and Fig. S6B), which pointed to the cellular target of faropenem as being important for the maintenance of rod shape during polar growth. In agreement, time-lapse microscopy of faropenem-treated cells revealed a loss of rod shape preferentially in the growth pole compartment. Remarkably, following cell division, the cell generated from the former growth pole compartment is large and round, whereas the other cell retains its rod shape (Fig. 5B and Movie S3). This phenotype was distinct from that associated with the treatment of the other carbapenem antibiotics and indicated that the cellular target(s) of faropenem was important to maintain proper PG integrity in the growth pole compartment.

**FIG 6:**
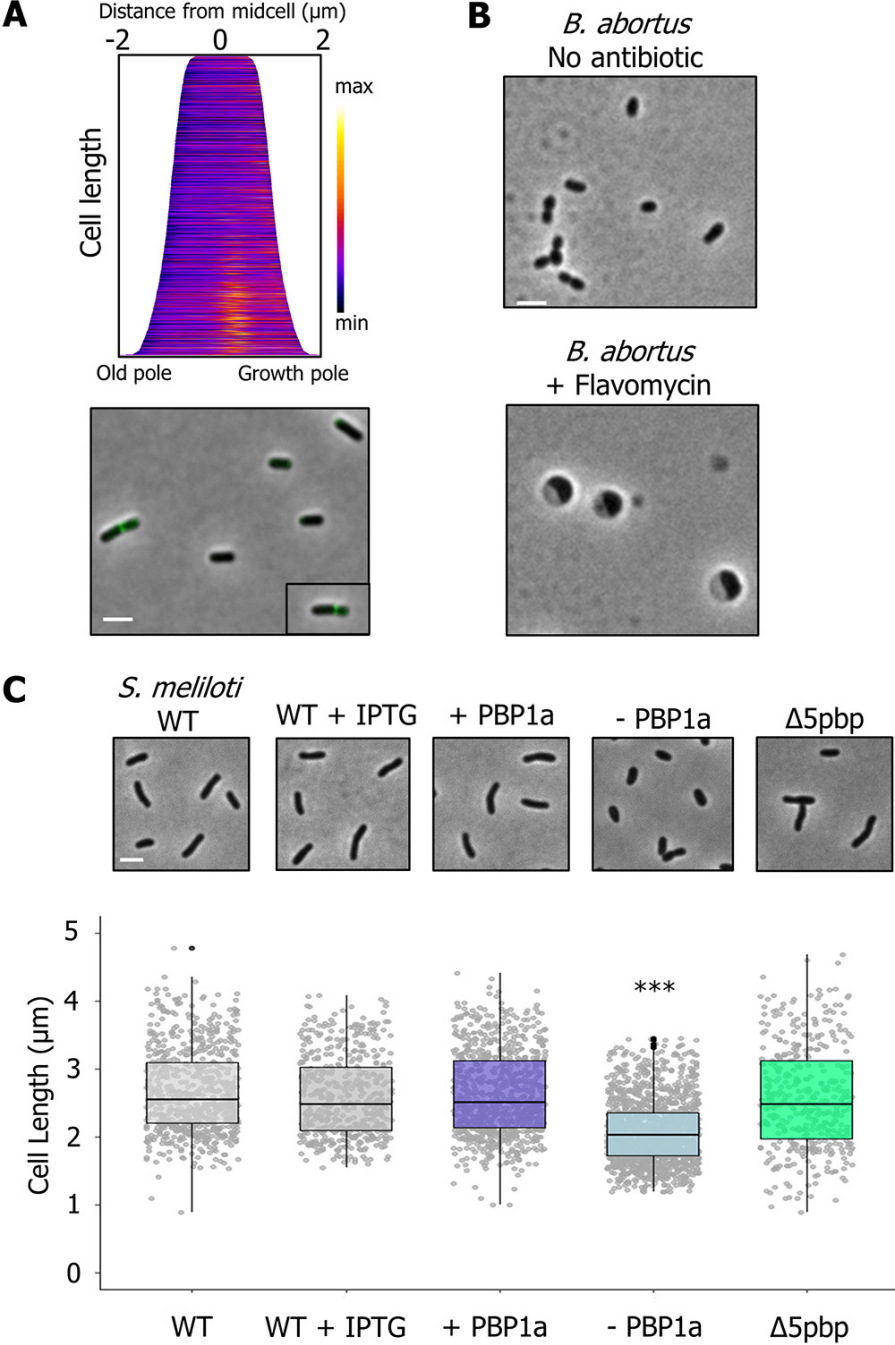
Mechanisms of polar growth are conserved in the *Rhizobiales* Brucella and *Sinorhizobium.* (A) Demograph and representative images of cells labeled with BADA–DA following treatment with 20 μg/ml flavomycin for 2 h; *n* = >300 per strain. Scale bar = 2 μm. (B) Representative phase microscopy images of B. abortus grown overnight without antibiotic or with 20 μg/ml of flavomycin. After 16 h, cells were spotted on a 1% Brucella broth agarose pad and imaged. (C) (Top) Phase microscopy images showing the phenotypes of S. meliloti wild-type (WT) and PBP mutants. Each strain was grown to exponential phase, spotted on a 1% TY agarose pad, and imaged by phase microscopy. (Bottom) Cell length distributions of PG synthase mutants. The indicated strains were grown to exponential phase, spotted on an agarose pad, imaged by phase microscopy, and subjected to cell length measurements using MicrobeJ (73). The data are shown as box plots in the style of Tukey (75) as described in the legend for Fig. 1. Distributions of cells significantly different from WT are indicated (***, one-way ANOVA with Bonferroni correction, *P* < 2 · 10^16^). *n* = >400 per strain.

10.1128/mBio.02346-21.7 FIG S6 Carbapenem and penem antibiotics impact the morphology of A. tumefaciens, and PBP1a is essential in *Sinorhizobium*. (A) Representative images of wild-type cells grown in the presence of 1.5 μg/ml imipenem, doripenem, and ertapenem and 50 μg/ml tebipenem. Cells were incubated with antibiotics for 24 h and then spotted on a 1% agarose pad and imaged using DIC microscopy. Scale bar = 2 μm. (B) Quantitative analysis and representative image of morphological changes following treatment of wild-type cells with 1.5 μg/ml faropenem for 24 h. The indicated strains were grown to the exponential phase, spotted on an agarose pad, imaged by phase microscopy, and subjected to cell length measurements using MicrobeJ. The data are shown as box plots in the style of Tukey as described in the legend for Fig. 1. Distributions of cells significantly different from the wild type are indicated (***, one-way ANOVA with Bonferroni correction, *P* < 2 · 10^16^). *n* = >300 per strain. (C) Characterization of S. meliloti PBP strains. Spot assays for cell viability of PBP deletion and depletion strains of S. meliloti. Dilutions are indicated above each spot, and images are representative of three independent biological replicates. (D) Cell width distributions of all strains from Fig. 6B. The indicated strains were subjected to cell width measurements using MicrobeJ (73). The data are shown as box and whisker plots in the style of Tukey (75). *n* = >400 per strain. Download FIG S6, TIF file, 1.4 MB. Copyright © 2021 Williams et al. 2021 Williams et al. https://creativecommons.org/licenses/by/4.0/ This content is distributed under the terms of the Creative Commons Attribution 4.0 International license.

10.1128/mBio.02346-21.10 MOVIE S3 Faropenem causes swelling of the growth pole. DIC image series of wild-type A. tumefaciens cells growing on a 1% ATGN agar pad supplemented with 1.5 μg/ml faropenem. Images were acquired every 10 min, and the movie is played at 10 frames per second for a total of 85 frames. Download Movie S3, AVI file, 0.3 MB. Copyright © 2021 Williams et al. 2021 Williams et al. https://creativecommons.org/licenses/by/4.0/ This content is distributed under the terms of the Creative Commons Attribution 4.0 International license.

To determine if the cellular target(s) of faropenem were conserved in other *Rhizobiales* we treated the closely related plant symbiont S. meliloti and the obligate intracellular pathogen B. abortus with sublethal concentrations of faropenem. We observed swelling of the growth pole in A. tumefaciens, S. meliloti, and B. abortus (Fig. 5D). Thus, we have identified the β-lactam antibiotic faropenem as a specific antibiotic inhibitor of polar growth among the *Rhizobiales*. Altogether, these data suggest that cell wall enzymes that are important for polar growth have a conserved role in agriculturally and medially important species of *Rhizobiales*.

### The essentiality of PBP1a is conserved among the *Rhizobiales*.

Since faropenem targeting of the growth pole machinery is conserved, we sought to determine if the essential role of PBP1a is also conserved in other *Rhizobiales*. We found that a 2-h treatment of A. tumefaciens with the PBP1a-specific GTase inhibitor flavomycin (moenomycin) (47) prevents polar growth as evidenced by formation of division sites closer to the new pole since there is less polar PG synthesis and the absence of polar labeling with NADA–DA (Fig. 6A). Since we showed that FDAADs are incorporated at the growth pole specifically by PBP1a (Fig. 3D and Fig. S3A and B), these data suggest flavomycin targets PBP1a in A. tumefaciens. In. B. abortus, flavomycin treatment causes growth arrest and the formation of large, round cells (Fig. 6B). A transposon mutagenesis screen of B. abortus predicted that out of the bifunctional PBPs, only PBP1a may be essential for growth (48), and Bandara and colleagues were unable to obtain a PBP1a mutant in Brucella melitensis, indicating that PBP1a is likely essential for viability (49).

Next, we explored the function of PBPs in the closely related plant symbiont S. meliloti. S. meliloti encodes six bifunctional PBP homologs, including one PBP1a homolog, four PBP1b homologs, and one PBP1c homolog. We constructed a Δ5pbp mutant, which is lacking all four PBP1b homologs and the PBP1c homolog and remains viable (see Fig. S6C). The Δ5pbp mutant had a median cell length similar to that of wild-type S. meliloti, but with a slightly broader distribution of cell lengths, with ∼88% of cells falling between 1.5 and 4 μm compared to ∼97% of WT cells falling between these cell lengths. Interestingly, the Δ5pbp mutant retained proper cell width (see Fig. S6D) and rod shape, similar to the Δ3pbp mutant of A. tumefaciens, suggesting that these enzymes contribute minimally to sustaining proper rod shape under standard growth conditions.

Similar to our findings for A. tumefaciens, we were unable to make a deletion of *pbp1a*, so we constructed a PBP1a depletion strain. Depletion of PBP1a led to a severe viability defect (see Fig. S6C), and cells became shorter (Fig. 6B) and wider (see Fig. S6D). Thus, PBP1a is essential for polar growth and maintenance of proper rod shape in both S. meliloti and A. tumefaciens. Taken together, our data from B. abortus, S. meliloti, and A. tumefaciens support the notion that PBP1a plays an essential and conserved role in polar growth among the *Rhizobiales*.

## DISCUSSION

**FIG 7:**
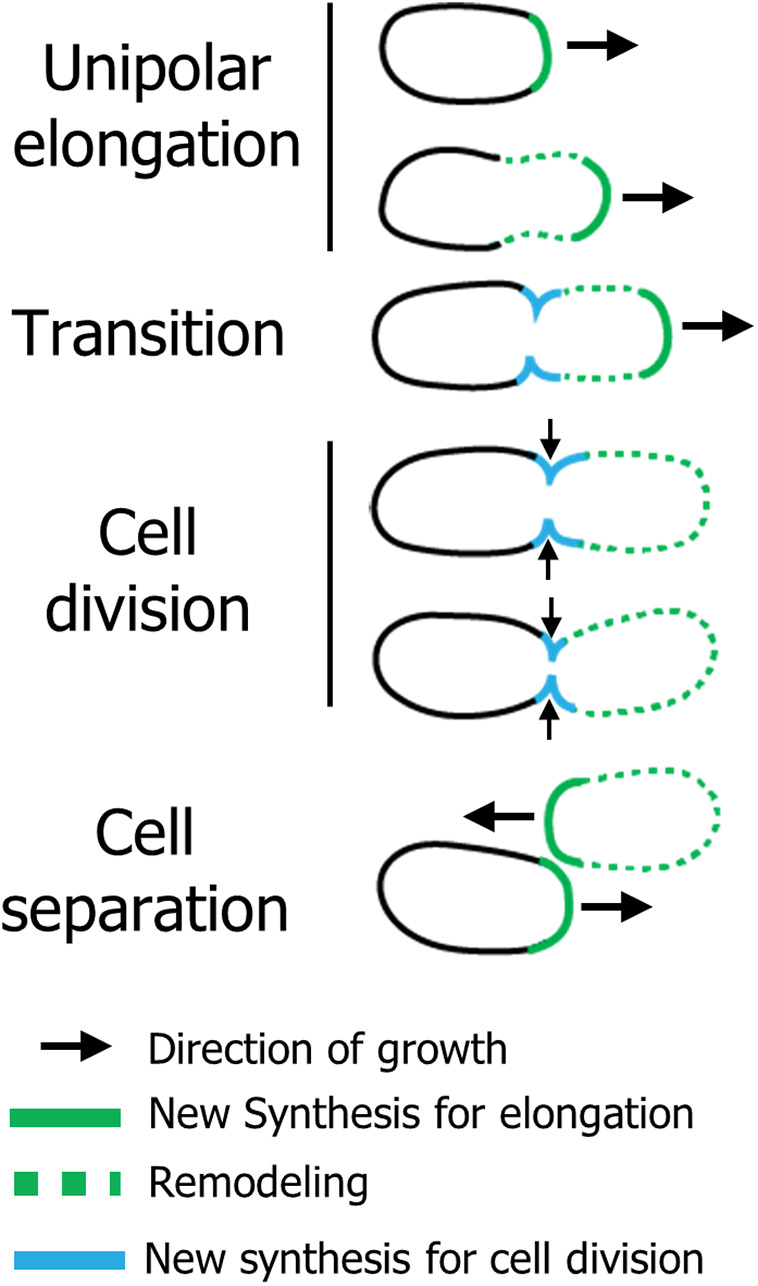
Model of cell wall synthesis in the *Rhizobiales*. (Unipolar elongation) A. tumefaciens elongates from a single pole using PBP1a, an essential class A PBP homolog. As the cell gets longer, the sidewalls of the new pole begin to be remodeled in a growth-independent manner through the activity of ld-transpeptidases. (Transition) Prior to cell division, growth at the pole is terminated and growth at the midcell is initiated, while remodeling of the new pole compartment continues. (Cell division) During cell division, new PG is added at the midcell through the transpeptidase activity of two class B PBPs (with PBP3a being the primary synthase) and the glycosyltransferase FtsW. (Cell separation) After cell separation, the new poles resume polar elongation from what was the site of cell division. Continuous remodeling of the new pole compartment occurs throughout the cell cycle.

Bacteria employ widely diverse growth strategies. Unipolar growth, or incorporation of new cell wall material at a single pole, is a shared mode of growth among the *Rhizobiales*, but the mechanisms that drive polar PG insertion remain poorly understood. Most bacteria have multiple class A PBPs with semiredundant functions, and growth is supported by the presence of any one of the PBPs (50). In Caulobacter crescentus, four class A PBPs can support growth when expressed alone (51). In B. subtilis, all four class A PBPs are dispensable, and the SEDS protein RodA has likely taken over the essential function of the class A PBPs during elongation (52). Current findings support the proposal that in laterally growing bacterial species, PBP1a homologs act as autonomous entities involved in PG remodeling or repair and do not function as a part of the core elongation machinery (10, 17, 18). Instead, the monofunctional glycosyltransferase RodA and the monofunctional transpeptidase PBP2 are required to maintain rod shape (10, 16). Even in A. tumefaciens, weak polar labeling of Bocillin-FL and a citrine-PBP1a fusion led to the hypothesis that PBP1a is not a major factor in polar PG synthesis (19). In contrast, here, we find that PBP1a is essential for polar elongation of A. tumefaciens and other *Rhizobiales* (Fig. 7). Loss of class A PBPs in E. coli or B. subtilis led to a decrease in cell width (53, 54). Conversely, depletion of PBP1a in A. tumefaciens and S. meliloti led to a significant decrease in cell length and an increase in cell width (Fig. 1B and C and Fig. S1B). This indicates that cells lacking PBP1a have a shorter period of cell elongation, and as a result, likely spend more time synthesizing the septum (Fig. 3C and Fig. S3A and B). Thus, the regulation of PG synthesis that governs cell length and cell width in the *Rhizobiales* utilizes a novel mechanism compared to well-studied model bacteria. Perhaps because class A PBPs function independently of cytoskeletal complexes (10), PBP1a was freely available to assume the role of the primary enzyme driving polar PG synthesis prior to the loss of the *mre* operon. Notably, the other bifunctional PBPs minimally contributed to the maintenance of proper rod shape under the conditions tested in A. tumefaciens and S. meliloti. However, it is likely that the additional class A PBPs may make dedicated contributions under specific growth conditions. For example, in E. coli, PBP1a homologs are required for optimal growth in alkaline pH, while PBP1b homologs are required under acidic conditions (55). Since the plant rhizosphere is an acidic environment (56), it is possible that the remaining PBPs have specialized functions to maintain growth when bacteria are associated with a plant host. Additionally, duplication of the monofunctional PBP3 homolog among the *Rhizobiales* is restricted to a few species that interact with plants, suggesting this is not a broad solution to the loss of PBP2, but that PBP3b homologs may contribute more significantly to bacterial growth *in plantae*. In agreement with this idea, we found that of the two monofunctional PBP homologs (PBP3a, a clear homolog of the division-specific FtsI, and PBP3b), PBP3a contributes to cell division, while deletion of *pbp3b* plays a minimal role in maintaining proper cell growth or cell shape under standard laboratory conditions (described also in reference 19) but forms a synthetic lethal pair with PBP3a that functions in cell division.

In laterally growing bacterial species, the role of scaffolding PG synthase enzymes during elongation is fulfilled in part by the actin homolog MreB (57). Homologs of MreBCD are absent in the *Rhizobiales*; thus, how PBP1a is recruited to the growth pole and how its activity is regulated remain unexplored. Recently, GPR (for growth pole ring), a large (∼226 kDa) apolipoprotein with similarity to the polar organizing protein TipN from C. crescentus, was reported to form a ring at the growth pole in A. tumefaciens, with depletion of this protein leading to rounded cells (58). This phenotype implicates GPR as a possible candidate to scaffold PG enzymes during elongation. In addition, PG synthesis by PBP1a also requires hydrolysis of the existing sacculus to allow for insertion of new muropeptides. A dd-endopeptidase (RgsM) that is predicted to have hydrolysis activity was recently shown to be essential for polar growth in S. meliloti (59), and thus represents an interesting candidate for polar PG hydrolysis.

Expanding our knowledge of the incorporation mechanisms of FDAA and FDAAD probes is critical for proper data interpretation of such labeling experiments and to unveil the molecular mechanisms of PG biosynthesis utilized by diverse bacteria. Several lines of evidence suggest that FDAAs are primarily incorporated into the cell wall extracellularly through remodeling by LDTs (20, 39, 42, 60, 61). In contrast, DAAD probes are incorporated into peptidoglycan precursors *in vitro* (6, 35). Additional evidence of cytoplasmic incorporation of DAADs includes bypass of d-cycloserine treatment (34) and rescue of mutants that fail to synthesize d-ala-d-ala dipeptides by endogenous addition of DAAD probes (35). Thus, DAAD-based probes are initially covalently linked to the PG precursor in the cytoplasm and subsequently incorporated into nascent PG by bifunctional PBPs. Consistently, our results indicate that, in A. tumefaciens, FDAAD probes report on nascent PG synthesis at the pole by PBP1a, whereas FDAA probes are incorporated by the localized activity of ld-transpeptidases on the bacterial cell surface. Given that fosfomycin treatment partially inhibited HADA incorporation (Fig. 3C), it suggests that some LDT activity in A. tumefaciens is growth dependent, while a portion of LDT activity is also growth independent, as seen by continued incorporation of HADA after fosfomycin treatment (see Fig. S2F) and along the sidewall of the growth pole compartment during incubation in nutrientless conditions (6). Functional specificity of a subset of LDTs during polar growth is further supported by the observation that Atu0845-sfGFP localized exclusively to the growth pole in wild-type cells (19) and was seen trapped in the growth poles of cells depleted of FtsZ (31). One possibility is that distinct LDTs contribute to insertion of nascent PG to promote lengthening, whereas others may modify the existing PG to widen the cell. Similar to our observation that HADA labels WT cells along the sidewalls of the new pole compartment much more brightly than the old pole compartment (Fig. 4A), using a clickable-DAA probe (alkDala), it was shown that sidewall labeling of the new pole compartment increases as the cell lengthens (19). Given the linear relationship between the increase in length and width of the new pole compartment (1, 19), it is possible that LDT activity along the sidewalls may contribute to widening of the growth pole compartment (Fig. 7). Additional studies will be required to determine if the observed change in circumference of the growth pole compartment represents an additional growth phase in A. tumefaciens and related bacteria.

Since LDTs are resistant to most classes of β-lactam antibiotics (62), cross-linking the cell wall via LDTs may contribute to the high antibiotic resistance to β-lactam antibiotics in the *Rhizobiales*. Here, we show that meropenem and faropenem inhibit ld- and dd-transpeptidation in A. tumefaciens, indicating that they may target LDTs and/or PBPs. Perhaps the loss of LDT activity disrupts the activity of high-molecular-weight PBPs if they function together in a growth pole complex. A combination of LDT- and PBP-targeting antibiotics acted synergistically in killing Mycobacterium tuberculosis (60), and a similar approach may also be effective against species in the *Rhizobiales*. Since faropenem causes swelling of the growth pole in A. tumefaciens, S. meliloti, and B. abortus it is likely that the target(s) of this drug are conserved components of the growth machinery. Identification of the specific cellular target(s) of faropenem will provide candidate proteins for further characterization.

Expanding our understanding of the mechanism of polar growth in the *Rhizobiales* will help to shed light on the different strategies that can be employed by bacteria during cell elongation. Indeed, our findings highlight intriguing parallels with other, distantly related, polarly growing bacteria, including the *Actinobacteria*. For example, PBP1a is essential in Mycobacterium smegmatis (63), an actinobacterium that grows by bipolar elongation. In addition, PBP1a localizes to growth poles and is important for maintenance of rod shape in other *Actinobacteria* (64, 65). Recently, a study of the PG synthases in Corynebacterium glutamicum, which lacks homologs to nearly all Rod complex proteins but encodes RodA-PBP2 and two class A PBPs (known as PonA and PonB), found that either the class A PBPs or RodA alone can sustain polar elongation (66). Deletion of the two class A PBPs had a more pronounced defect in cell length, width, and viability than the RodA mutant, and overproduction of PonB (PBP1b) was able to restore the cell viability defect of the Δ*rodA*Δ*ponA* mutant, suggesting that the class A PBPs play a major role in proper polar cell wall synthesis. Localization studies and labeling of the PG synthase mutants with clickable d-amino acid dipeptides revealed that RodA and class A PBPs both contribute to polar PG insertion but that the class A PBPs likely contribute to a broader region of cell wall insertion that extends beyond the poles, reiterating the usefulness of dipeptide probes for investigation of cell wall synthesis in polar-growing species (66). The reliance on class A PBPs for synthesis of PG at the pole may be a key feature of polar-growing bacteria that arose independently in the *Rhizobiales* and *Actinobacteria*, indicating convergent evolution. Notably, the cell wall of polar-growing bacteria in both clades also contains a high proportion (∼30 to 80%) of LDT-cross-linked PG (1, 67, 68), suggesting that ld-cross-links may provide structural integrity. Furthermore, in Mycobacterium, deletion of LDT-encoding genes leads to a loss of rod shape (62), and LDTs also contribute to active PG synthesis of the sidewalls (35). Finally, carbapenem antibiotics are routinely used to treat M. tuberculosis infections (22), hinting that the target of these drugs may be important in polar-growing bacteria. Future work directed at characterizing the role of LDTs during polar growth in *Rhizobiales* and *Actinobacteria* is needed to determine if the high degree of ld-cross-linking is an innovation which allows for polar elongation to be adopted as the primary mode of growth. Overall, the possibility that there may be governing principles which allow for polar growth to emerge as a successful growth strategy is a fascinating concept which merits further study.

## MATERIALS AND METHODS

### Bacterial strains, plasmids, and growth conditions.

A list of all bacterial strains and plasmids used in this study is provided in Text S1 in the supplemental material. Agrobacterium tumefaciens C58 and derived strains were grown in ATGN minimal medium (69) without exogenous iron at 28°C with shaking. When appropriate, kanamycin (KAN) was used at the working concentration of 300 μg/ml. When indicated, isopropyl β-d-1-thio-galactopyranoside (IPTG) was used as an inducer at a concentration of 1 mM. Sinorhizobium meliloti strains were grown in tryptone-yeast (TY) medium. When appropriate, KAN was used at the working concentration of 100 μg/ml, gentamicin (GM) was used at 20 μg/ml, and IPTG was used at a concentration of 500 μg/ml. Brucella abortus strain S19 was grown in Brucella broth. E. coli strains were grown in Luria-Bertani medium at 37°C. For E. coli DH5α and S17-1 λ *pir*, when appropriate, 50 μg/ml or 30 μg/ml of KAN, respectively, was added.

10.1128/mBio.02346-21.1 TEXT S1 [Database] Supplemental text with strain and plasmid lists and supplemental materials and methods. Download Text S1, DOCX file, 0.3 MB. Copyright © 2021 Williams et al. 2021 Williams et al. https://creativecommons.org/licenses/by/4.0/ This content is distributed under the terms of the Creative Commons Attribution 4.0 International license.

### Construction of strains and plasmids.

A list of all primers used in this study is provided in Text S1. For amplification of target genes, primer names indicate the primer orientation and added restriction sites. All expression vectors were verified by sequencing. All vectors were introduced into A. tumefaciens strains utilizing standard electroporation protocols (70), with the addition of IPTG in the medium when introducing plasmids into depletion backgrounds.

### Construction of deletion/depletion plasmids and strains.

Vectors for gene deletion by allelic exchange were constructed using recommended methods for A. tumefaciens (70). Briefly, 500-bp fragments upstream and 500 bp downstream of the target gene were amplified using primer pairs P1/P2 and P3/4, respectively. Amplicons were spliced together by splicing by overlap-extension (SOEing) using primer pair P1/P4. The amplicon was digested and ligated into pNTPS139. The deletion plasmids were introduced into A. tumefaciens by mating using an E. coli S17 conjugation strain to create KM-resistant, sucrose-sensitive primary integrants. Primary integrants were grown overnight in medium with no selection. Secondary recombinants were screened by patching for sucrose resistance and KM sensitivity. Colony PCR with primers P5/P6 for the respective gene target was used to confirm deletion. PCR products from P5/P6 primer sets were sequenced to further confirm deletions.

For depletion strain construction, target genes (*pbp1a* or *pbp3a*) were amplified, digested, and ligated into pUC18-mini-Tn*7*T-GM-P_lac_. The mini-Tn*7* vector, along with the pTNS3 helper plasmid, were introduced into C58Δ*tetRA*::a-*att*Tn*7* as described previously (26). Transformants were selected for GM resistance, and insertion of the target gene into the a-*att* site was verified by colony PCR using the tet forward and Tn7R109 primer. PCR products were sequenced to confirm insertion of the correct gene. Next, the target gene was deleted from the native locus as described above in the presence of 1 mM IPTG to drive expression of the target gene from the engineered site.

To generate the S. meliloti strain lacking the five nonessential PBPs, the corresponding genes were consecutively deleted from the Rm2011 *rgsP-egfp* genome using the sucrose selection method (71). To generate the S. meliloti MrcA1 depletion strain, first, plasmid pK18mobsac-mrcA1del was integrated into the Rm2011 *rgsP-egfp* genome, and then an ectopic *mrcA1* copy was introduced on plasmid pGCH14-mrcA1, followed by sucrose selection of mutant clones with deletion of the native *mrcA1* allele. The curable plasmid pGCH14, which is maintained in a single copy in S. meliloti due to the replication operon *repABCpMlb_lacO*, prone to repression by LacI was used as the vector to conditionally establish the ectopic copy of *mrcA1* under the control of its native promoter. pSRKKm, carrying *lacI*, was introduced into the strain with chromosomal deletion of *mrcA1*, carrying pGCH14-mrcA1, and the resulting strain Rm2011 *rgsP-egfp mrcA1*^dpl^ was grown in the presence of 500 μm IPTG. Growth in the absence of IPTG induced MrcA1 depletion due to loss of the ectopic *mrcA1* copy.

### Phase and fluorescence microscopy.

A small volume (∼1 μl) of cells in the exponential phase (optical density at 60 nm [OD_600_], 0.2 to 0.4) was applied to a 1% ATGN agarose pad as described previously (72). Differential interference contrast (DIC), phase contrast, and epifluorescence microscopy were performed with an inverted Nikon Eclipse TiE and a QImaging Rolera em-c2 123 1K EMCCD camera with Nikon Elements Imaging Software. For time-lapse microscopy, images were collected every 10 min, unless otherwise stated.

### Quantification of cell length distributions.

Cells were grown overnight in ATGN. Cells were diluted in ATGN to an OD_600_ of 0.2 and allowed to grow until reaching an OD_600_ of 0.4 to 0.6. Live cells were imaged using phase-contrast microscopy, and cell length distributions of the indicated number of cells per strain were determined using the longest medial axis as measured using MicrobeJ software (73).

### Quantification of cell morphologies, FDAA, and FDAAD labeling patterns.

Methods for the synthesis of fluorescent d-amino acid dipeptides are included in Text S1. For A. tumefaciens, cells were grown overnight in ATGN medium and diluted under the same conditions to an OD_600_ of 0.20 and allowed to grow until reaching an OD_600_ of 0.4 to 0.6. At this point, cells were labeled with 1 mM fluorescent d-amino acid (FDAA) HCC-amino-d-alanine (HADA), or the fluorescent d-amino acid dipeptide (FDAAD) NBD-amino-d-alanine-d-alanine (HADA–DA) as previously described (34, 36, 39). Immediately following a 5-min incubation (Fig. 3, 4, and 6 and Fig. S2E and S3A and B) or a 60-min incubation (Fig. S4C), cells were ethanol-fixed to prevent further growth. Phase-contrast and epifluorescence microscopy was performed on the reported number of cells. For Fig. 3C and Fig. S2F, A. tumefaciens cells were incubated for 1 h with 30 mM fosfomycin prior to labeling. For Fig. 6A, A. tumefaciens cells were incubated for 2 h with 15 μg/ml of flavomycin prior to HADA–DA labeling. For E. coli, cells were grown overnight at 37°C in M9 + 0.2% glucose minimal medium and diluted under the same conditions to an OD_600_ of 0.1 and allowed to grow until reaching an OD_600_ of 0.4 to 0.6. At this point, cells were labeled with 1 mM HADA–DA. After an incubation of 90 min, cells were ethanol-fixed and washed 3 times in 1 ml phosphate-buffered saline (PBS) before imaging. For B. subtilis 3610, cells were grown overnight at 37°C in S750 + 1% glucose defined minimal medium and diluted under the same conditions to an OD_600_ of 0.1 and allowed to grow until reaching an OD_600_ of 0.4 to 0.6. At this point, cells were labeled with 5 mM HADA–DA. After an incubation of 120 min, cells were ethanol-fixed and washed 3 times in 1 ml PBS before imaging. For *S. venezuelae*, cells were grown overnight at 30°C in LB medium and diluted under the same conditions to an OD_600_ of 0.1 and allowed to grow until reaching an OD_600_ of 0.4 to 0.6. At this point, cells were labeled with 1 mM fluorescent boron-dipyrromethene (BODIPY FL)-amino-d-alaninyl-d-alanine (BADA–DA). After an incubation of 15 min, cells were washed once with 1 ml LB and resuspended in 500 μl LB containing 2 mM HADA–DA. After an incubation of 15 min, cells were washed once with 1 ml LB and resuspended in 500 μl LB containing 1 mM ATTO 610-amino-d-alaninyl-d-alanine (Atto610ADA–DA). After an incubation of 15 min cells were ethanol-fixed and washed 3 times in 1 ml PBS before imaging.

Demographs were constructed using MicrobeJ. For demographs, cells were arranged from top to bottom according to their cell lengths, and each cell was oriented such that the new pole (defined as the cell pole with the higher fluorescence intensity as determined by FDAA or FDAAD labeling or the smaller pole diameter in cells without label) was oriented to the right.

### PG compositional analysis.

For PG analysis, three cultures of each of strain were grown overnight in 3-ml culture tubes of ATGN minimal medium at 28°C with shaking; the + PBP1a strain was supplemented with 1 mM IPTG. The 3-ml cultures were then added to 50-ml flasks of fresh ATGN and allowed to grow under the same conditions until reaching an exponential-phase OD_600_ of 0.5 to 0.6. Cells were then pelleted by centrifugation at 4,000 × *g* for 10 min. Cell pellets were washed three times with ATGN by centrifugation and resuspension to remove IPTG. After the final wash, the 3 cell pellets from the + PBP1a strain were split and resuspended in 50 ml ATGN with or without IPTG. Each culture was grown for 16 h to an OD_600_ of 0.6 After 146 h of growth, 50 ml of the exponential cultures was collected and pelleted by centrifugation at 4,000 × *g* for 20 min. Cell pellets were resuspended in 3 ml of ATGN and 6 ml of 6% SDS and stirred with magnets while boiling for 4 h. Next, samples were removed from heat but continued to be stirred overnight. Samples were then shipped to the Cava laboratory for purification and analysis. Upon arrival, cells were boiled and simultaneously stirred by magnets for 2 h. After 2 h, boiling was stopped, and samples were stirred overnight. PG was pelleted by centrifugation for 13 min at 60,000 rpm (TLA100.3 Beckman rotor, Optima Max-TL ultracentrifuge; Beckman), and the pellets were washed 3 to 4 times by repeated cycles of centrifugation and resuspension in water. The pellet from the final wash was resuspended in 50 μl of 50 mM sodium phosphate buffer, pH 4.9, and digested overnight with 100 μg/ml of muramidase at 37°C. Muramidase digestion was stopped by boiling for 4 min. Coagulated protein was removed by centrifugation for 15 min at 15,000 rpm in a desktop microcentrifuge. The muropeptides were mixed with 15 μl 0.5 M sodium borate and subjected to reduction of muramic acid residues into muramitol by sodium borohydride (10 mg/ml final concentration, 20 min at room temperature) treatment. Samples were adjusted to pH 3 to 4 with orthophosphoric acid and filtered (0.2-μm filters). Analysis of muropeptides was performed on an ACQUITY ultraperformance liquid chromatography (UPLC) BEH C_18_ column (130 Å, 1.7 μm, 2.1 mm by 150 mm; Water, USA) and detected at an absorbance of 204 nm with an ACQUITY UPLC UV-visible detector. For the data shown in Fig. 4B and Fig. S5B, muropeptides were separated with organic buffers at 45°C using a linear gradient from buffer A (formic acid, 0.1% [vol/vol] in water) to buffer B (formic acid, 0.1% [vol/vol] in acetonitrile) in an 18-min run with a 0.25 ml/min flow. For the data shown in Fig. 5A and Fig. S5A and C, muropeptides were separated using a linear gradient from buffer A (sodium phosphate buffer, 50 mM; pH 4.35) to buffer B (sodium phosphate buffer, 50 mM; pH 4.95; methanol, 15% [vol/vol]) with a flow of 0.25 ml/min in a 20 min run. Individual muropeptides were quantified from their integrated areas using samples of known concentration as standards. Muropeptide abundance was statistically compared using a one-way analysis of variance (ANOVA) with Tukey’s multiple-comparison test.
