# Supplementary figures and images for: Unipolar Peptidoglycan Synthesis in the Rhizobiales Requires an Essential Class A Penicillin-Binding Protein
Source: mBio. 2021 Sep 21;12(5):e02346-21. doi: 10.1128/mBio.02346-21 (PMC8546619; doi:10.1128/mBio.02346-21)

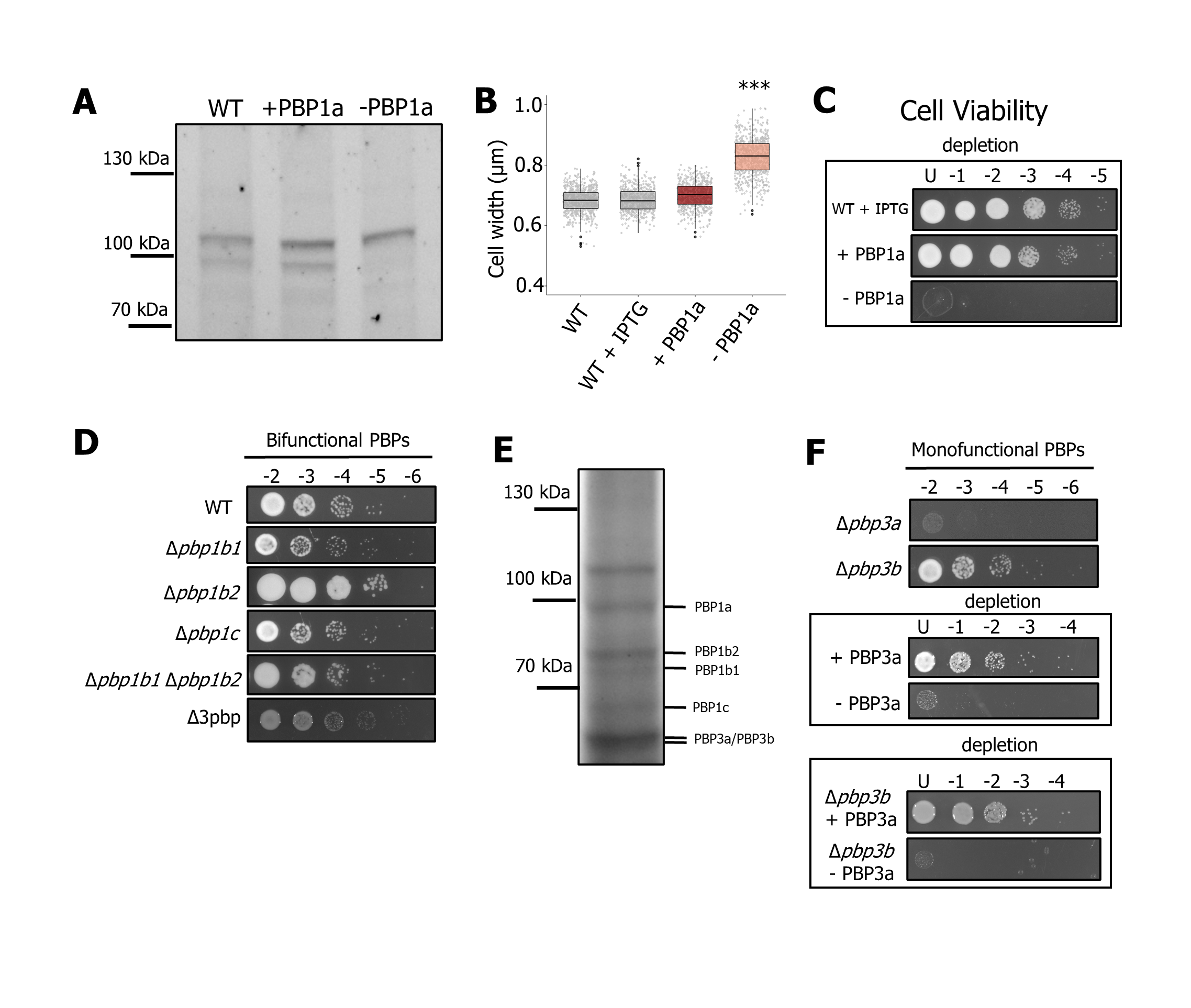

Supplement: FIG S1 [file mbio.02346-21-sf001.tif]

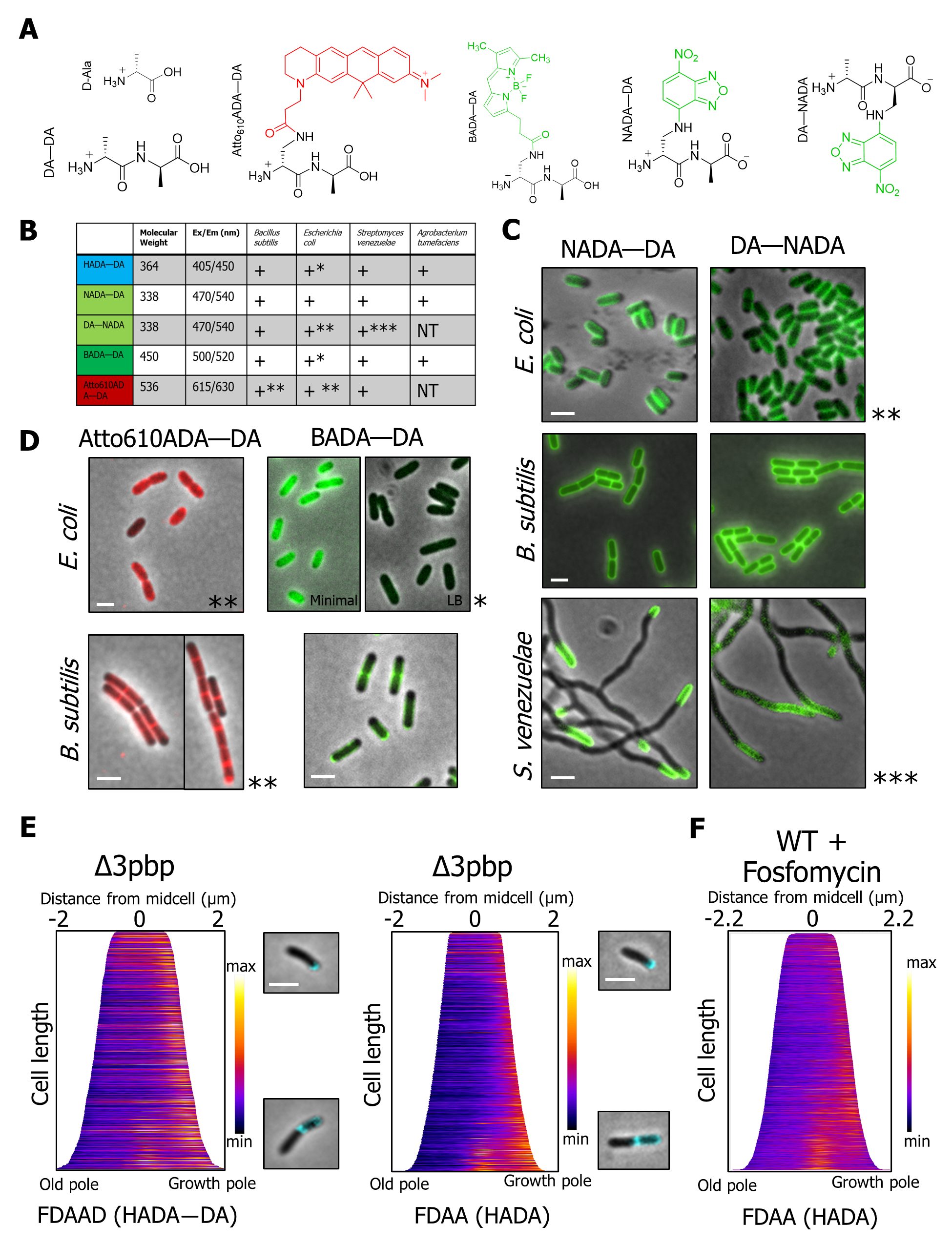

Supplement: FIG S2 [file mbio.02346-21-sf002.tif]

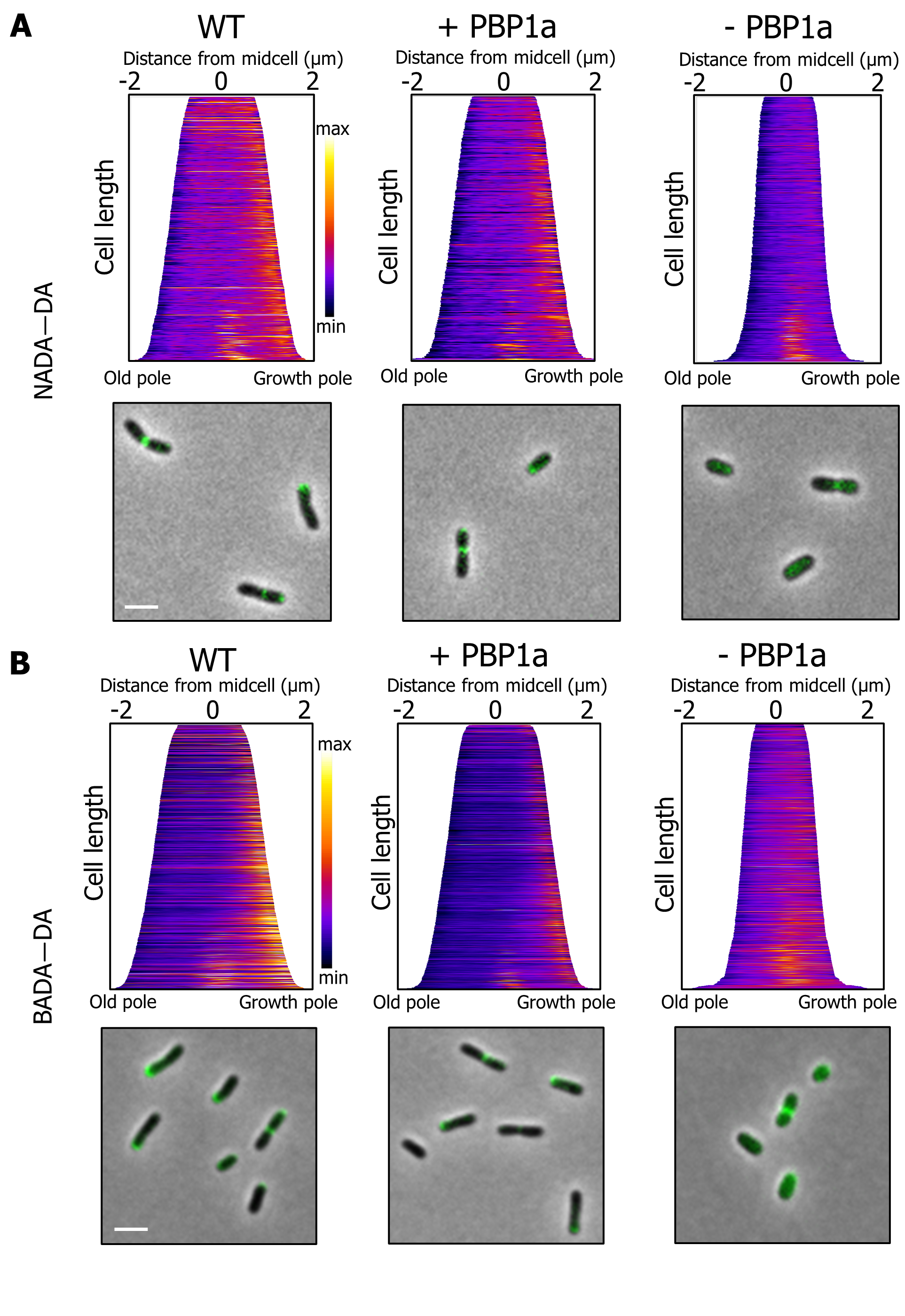

Supplement: FIG S3 [file mbio.02346-21-sf003.tif]

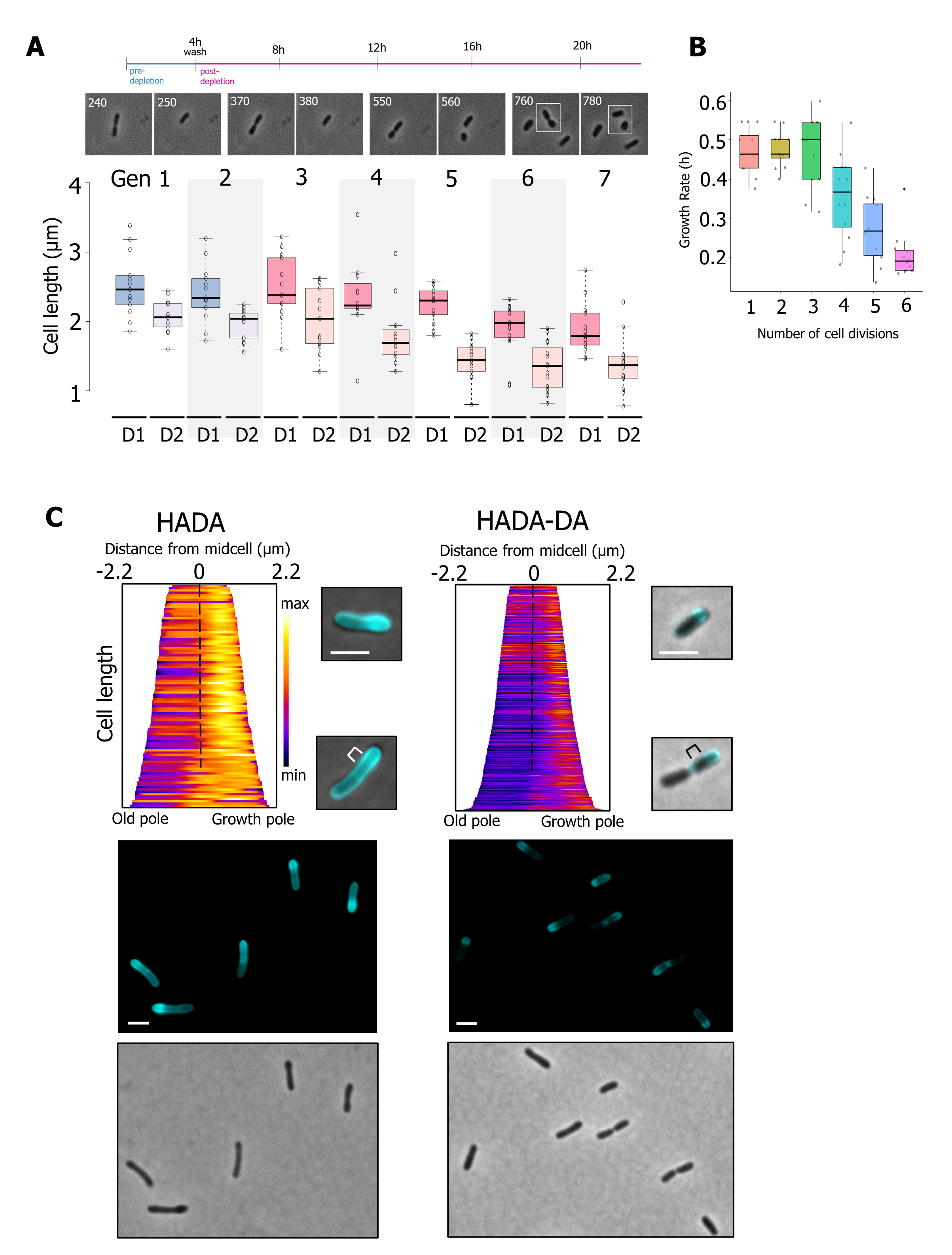

Supplement: FIG S4 [file mbio.02346-21-sf004.tif]

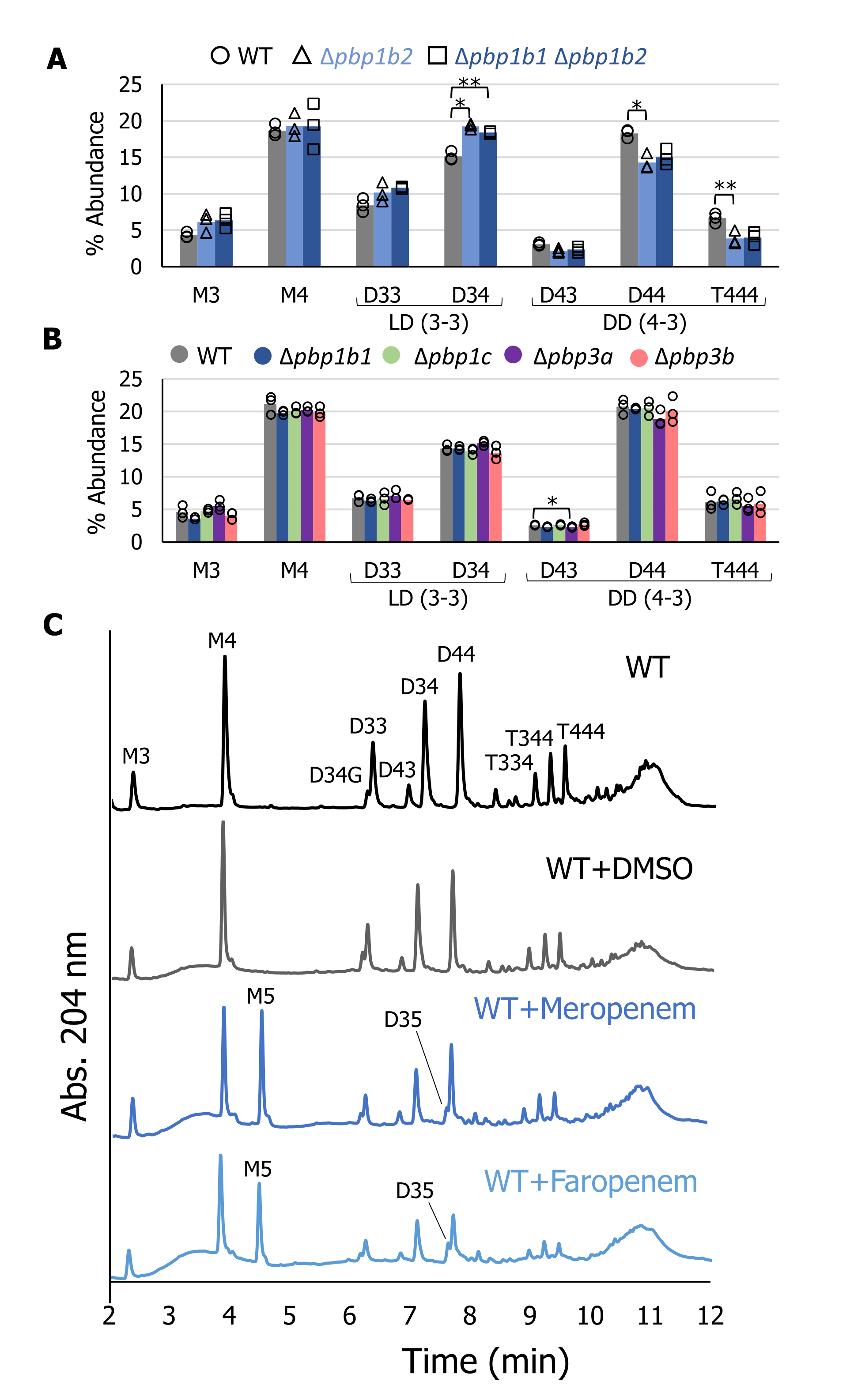

Supplement: FIG S5 [file mbio.02346-21-sf005.tif]

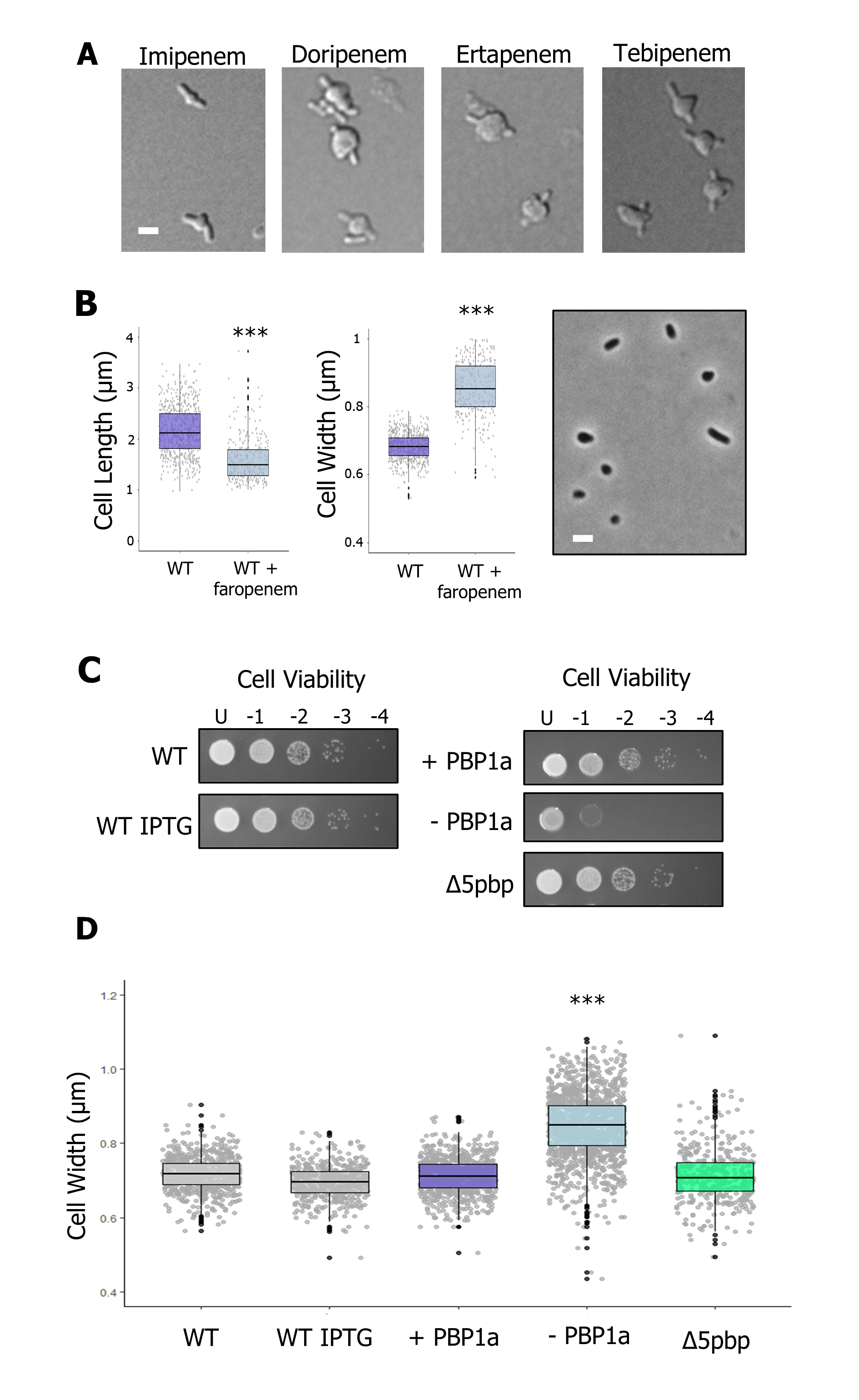

Supplement: FIG S6 [file mbio.02346-21-sf006.tif]
